# Using Natural Language Processing to Identify Adverse Drug Events Characterized by Medication Replacement in Primary Care Electronic Medical Records: Algorithm and Validation Study

**DOI:** 10.2196/86467

**Published:** 2026-08-10

**Authors:** Alan Katz, Abhishek Dhankar, Gillian Fransoo, Diane Gordon Pappas, Amani F Hamad, Christine Leong, Alexander Singer

**Affiliations:** 1 Manitoba Centre for Health Policy, College of Community and Global Health, University of Manitoba, Room 408-727 McDermot Ave, Winnipeg, MB, R3E 3P5, Canada, 1 204 955 2612; 2 Department of Family Medicine, Max Rady College of Medicine, University of Manitoba, Winnipeg, MB, Canada; 3 College of Community and Global Health, University of Manitoba, Winnipeg, MB, Canada; 4 Rady Faculty of Health Sciences, College of Pharmacy, University of Manitoba, Winnipeg, MB, Canada

**Keywords:** machine learning, natural language processing, adverse drug events, electronic medical records, large language model

## Abstract

**Background:**

Health care systems generate vast amounts of unstructured text, such as clinical notes, which capture nuanced patient experiences, clinical reasoning, and subtle indicators of health status. While health system research has traditionally relied upon structured data, natural language processing (NLP) enables the extraction of this rich textual information. Leveraging NLP could improve the identification and characterization of underreported adverse drug events (ADEs).

**Objective:**

The primary objective of this study was to train and evaluate multiple NLP models, including both previously published architectures and a novel model, for the identification of ADEs from clinical notes.

**Methods:**

Electronic medical records from the Manitoba Primary Care Research Network (MaPCReN) were used in this study. Clinical notes were annotated to indicate the presence of a possible ADE, the associated words or phrases, and the corresponding drug. A subselection algorithm was applied to ensure sufficient representation of notes containing ADEs for training a robust classifier. The cohort was restricted to patients aged 55 years and older and was annotated in 2 waves: Wave 1 comprised primary care encounter notes selected for a temporally linked emergency department (ED) visit, enriching it for acute presentations, while Wave 2 relaxed this requirement, and its acuity composition was uncharacterized. The annotated data were split into training and test sets. NLP models—including BioBERT, BlueBERT, a large language model (LLM) classifier, and an LLM embeddings–based classifier—were trained on both original clinical notes and notes reformatted into Subjective-Objective-Assessment-Plan (SOAP) structure. To approximate a clinician-inspired reasoning workflow, Mistral-7b-Instruct was used to extract presenting symptoms and generate a ranked list of potential etiologies. Model performance was evaluated using precision, recall, and *F*_1_-score.

**Results:**

Of the 1085 annotated encounter notes, 355 included ADEs. Across 9 modeling approaches evaluated over 5 random seeds, models trained on SOAP-rewritten notes generally outperformed those trained on original notes. The SOAP Rewrite+ LLM Embeddings Classifier (GritLM-SOAP) achieved the highest mean *F*_1_-score (72.53%; 95% CI 62.40%‐81.82%), while BioBERT-SOAP weighted achieved the highest mean recall (76.34%; 95% CI 62.69%‐89.66%). End-to-end span-level extraction (named entity recognition+relation extraction) on ADE-positive test notes achieved a mean relaxed *F*_1_-score of 0.455, with relation extraction identified as the bottleneck.

**Conclusions:**

Effective detection of ADEs in clinical notes may benefit from NLP models that approximate the clinical reasoning of health care providers. While the SOAP Rewrite+ LLM Embeddings Classifier demonstrated a reasonable balance of precision and recall, there is room for improvement as models evolve.

## Introduction

Natural language processing (NLP) is a branch of AI that allows computers to interpret human language. Health care systems and patients produce substantial unstructured text data such as clinical notes, academic papers, and posts on health forums and social media. NLP can leverage unstructured text data from these sources to automate time-consuming review and classification processes, enabling the extraction of relevant insights. Many potential applications of NLP in health care have been explored, including summarizing clinical notes, assisting in drug discovery, detecting depression through social media analysis, and detecting adverse drug events (ADEs) [1-15].

Historically, observational studies in health care have relied upon structured data, which has predetermined attributes. Unlike structured data, unstructured sources can include nuanced information about patient experiences, clinical reasoning, social and behavioral context, and subtle indicators of health status. Incorporating this information can significantly improve analytical models. For example, Soguero-Ruiz et al [16] showed that predictive models for colorectal surgical complications performed best when combining unstructured text and structured data, with unstructured text alone outperforming structured data.

Although the terms adverse drug reaction (ADR) and ADE are often used interchangeably in the literature, ADRs specifically refer to harmful or unpleasant effects that are causally linked to a drug taken at normal therapeutic doses as prescribed [17]. In contrast, ADEs encompass a broader range of adverse effects related to medication use, including those resulting from drug misuse, nonadherence, drug-drug interactions, and allergic reactions [18]. Thus, ADRs represent a subset of ADEs and, in this study, we use the term ADE to capture this wider scope of medication-related harm. ADRs are likely underreported, partly because reporting is typically voluntary for medical professionals, who may be indifferent or lack motivation to report suspected cases [19,20]. NLP can enhance identification and characterization of ADEs in the realms of postmarketing drug surveillance and continuous quality improvement by extracting valuable clinical information from unstructured data sources [21-23]. We excluded incorrect dosage administration, which may lead to an unexpected “over-response” to the drug, as these are generally administration errors rather than drug issues.

NLP research typically relies on domain experts who annotate data to establish “benchmark datasets,” which are then used to train and evaluate NLP models [24,25]. For instance, in its simplest form, a dataset for building an ADE detection model could consist of clinical notes that have been reviewed and annotated for whether they include an indication of an ADE. Such benchmark datasets provide a uniform basis for the comparison of models that have been built using the datasets. These datasets are often released as part of competitions, where teams compete to develop models that achieve the highest performance metrics [6,26].

Several benchmark challenges have contributed to advancing ADE detection, including foundational datasets such as MADE 1.0 and n2c2, and more recent efforts such as Social Media Mining for Health (SMM4H) shared tasks [6,11,26]. These initiatives have developed annotated datasets and evaluation tasks using unstructured clinical notes and social media text, providing standardized resources for named entity recognition (NER), a method for automatically identifying and labeling clinically relevant terms in free-text notes, and relation extraction (RE), a method for identifying relationships between terms related to medications and ADEs. While MADE 1.0 and n2c2 remain important historical benchmarks, newer datasets such as MultiADE and SMM4H 2025 have expanded domain diversity, linguistic coverage, and data scale [27,28]. Despite these advances, model performance on ADE detection tasks remains modest [6,11,26]. Common challenges include capturing long-range dependencies between drugs and ADEs that are far apart in a sentence or document, frequent misclassification of ADEs and indications, and heterogeneity across diverse data sources. These limitations underscore the ongoing need for NLP methods capable of more accurately identifying ADEs in complex clinical and social contexts.

Therefore, the objective of this study was to explore the potential of NLP models to identify ADEs in diverse clinical notes.

## Methods

### Study Design

Our study was designed as a 2-stage research plan. The first stage–reported in this paper–evaluates document-level binary classification to determine whether NLP models can reliably identify encounter notes containing ADEs. The second stage, contingent on achieving sufficient classification performance, would apply span-level extraction using large language models (LLMs) to identify the specific drug-symptom pairs within flagged notes.

### Data Source and Annotation

We used the Manitoba Population Research Data Repository (Repository), which houses an extract of the Manitoba Primary Care Research Network (MaPCReN), a subset of the Canadian Primary Care Sentinel Surveillance Network (CPCSSN) data [29]. The Repository includes electronic medical record (EMR) data from April 1, 1995 to December 31, 2021. Using Drug Program Information Network (DPIN) and emergency department (ED) hospital record data in the Repository, we created an annotated dataset of clinical notes, which were annotated to indicate whether they contained a possible ADE, the specific words or phrases associated with the ADE, and the corresponding drug. This dataset was used to train several NLP models previously described in the literature. The source dataset contains patient demographics, diagnoses, medications, allergies, and other clinical details for patients in Manitoba. The complete data repository holds approximately 2 million clinical notes reflecting primary care patient visits to primary care providers.

### Clinical Note Selection to Enrich Training Data

#### Medication Replacement Event Identification

##### Continuous Medication Span Construction

Medication replacement events were identified using pharmacy dispensing records from the structured data in the DPIN. Consecutive dispensations of the same Anatomical Therapeutic Chemical (ATC) code were merged into continuous medication spans. The end date of each dispensing was estimated by adding the days’ supply to the dispensing date. If a subsequent dispensing of the same medication occurred within 90 days of the estimated end date of the prior dispensing, the 2 were merged into a single continuous span. This 90-day grace period accounts for early refills, stockpiling, and minor gaps in adherence.

##### Therapeutic Class Grouping

Medications were grouped into therapeutic classes based on their ATC codes to enable within-class comparisons. The therapeutic classes included antihypertensives (ATC codes C02-C09), antidepressants (N06A), anxiolytics (N05BA and N03AE), antidiabetics (A10), lipid-modifying agents (C10), osteoporosis medications (M05B), and anti-infectives (J01 and G04A). All continuous medication spans for a given patient were indexed by both patient identifier and therapeutic class, enabling pairwise comparison of medications within the same class.

##### Replacement Event Detection

A replacement event was defined as the initiation of a new medication from the same therapeutic class within 30 days of the dispensing date of another medication in that class. Specifically, for 2 medications A and B belonging to the same therapeutic class but having different ATC codes, a replacement was flagged if medication B was first dispensed within 30 days after the dispensing date of medication A. Concurrent use of the 2 medications (ie, overlapping supply periods) did not preclude identification as a replacement event.

##### Exclusion Criteria

Potential ADEs related to drug dosage were addressed by excluding events in which the original medication was subsequently redispensed at any point after the replacement event, on the rationale that a medication switch driven by an ADE would be permanent; the patient would not return to the offending drug. Only replacement events from 2015 onward were retained to ensure availability of corresponding clinical documentation in the MaPCReN EMR system.

### Encounter Note Selection

#### Note Retrieval

For each identified replacement event, encounter notes from the MaPCReN EMR database were retrieved. Eligible notes included those created between the dispensing date of the original medication and the dispensing date of the replacement medication, where the note was associated with the same patient and the same ATC code as the original medication. This temporal window captures clinical documentation that may describe the rationale for the medication change, including symptoms or adverse effects that prompted the switch.

When multiple notes satisfied these criteria, the most recent note, the one closest in time to the replacement event, was selected. This note was assumed to be the most likely to contain documentation of the clinical reasoning behind the medication change.

The cohort was further restricted to patients aged ≥55 years at the time of the encounter note. This age threshold was applied as an inclusion criterion to enrich the cohort for patients more likely to experience polypharmacy-related ADEs. Polypharmacy is a well-established risk factor for ADEs, and its prevalence rises sharply through the late 50s as multimorbidity becomes increasingly common; the ≥55 years threshold captures the onset of this transition. The cohort accordingly contains no patients younger than 55 years and 5 patients aged 55‐59 (the lower edge of the eligible range).

#### Annotation Waves

The dataset was constructed in 2 sequential annotation waves. Both waves drew from the same underlying data source using identical annotation guidelines. Wave 1 used the replacement event identification criteria described above, with the additional requirement that the patient must have had an ED visit within 7 days prior to the creation of the encounter note. This ED visit linkage was performed by matching patient identifiers against hospital admission records from the Repository. This initial constraint was intended to enrich for notes documenting acute ADEs that may have precipitated an ED visit. Replacement events involving all therapeutic classes, including anti-infectives, were eligible in Wave 1.

Wave 2 relaxed the ED visit requirement, drawing from the broader pool of replacement events without requiring a temporally linked ED visit. An additional exclusion criterion was introduced in Wave 2: events involving anti-infective medications (ATC codes J01 and G04A) as the original medication was excluded, as switching between antibiotics is common clinical practice when an antibiotic does not appear to be effective and is less likely to reflect ADEs. Notes already included in Wave 1 were removed from the Wave 2 pool to prevent duplication.

**Figure 1.**
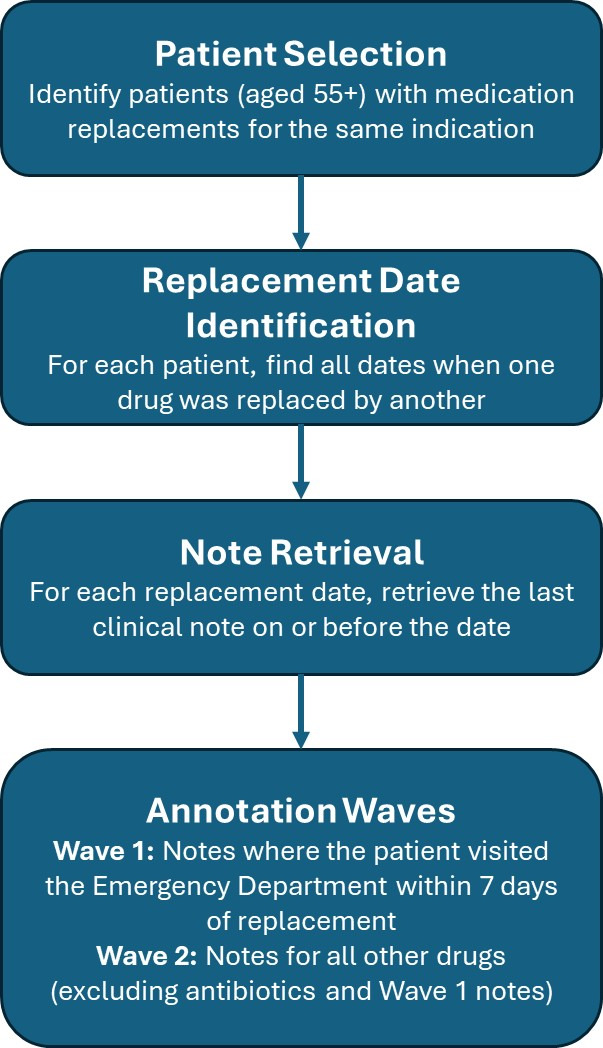
A flowchart summarizing the process used for identifying and annotating adverse drug events in clinical notes used in this study.

The final annotated dataset comprised 1085 encounter notes (Wave 1: 258; Wave 2: 827), of which 355 contained documentation of ADEs. A summary of the note selection and annotation is shown in Figure 1.

Both waves were initially identified using the same medication replacement algorithm but differed in their inclusion criteria as described above (Wave 1 ED-linked and Wave 2 with the ED requirement relaxed). The 2 waves’ annotation was conducted sequentially to manage annotator workload. The BRAT annotation software was used to annotate the extracted notes [30]. Two pharmacy student annotators used a collaborative annotation approach, reviewing notes together and reaching consensus on ambiguous cases rather than annotating independently. As such, formal interannotator agreement statistics were not calculated.

The collaborative approach ensured consistent application of annotation criteria and allowed real-time resolution of ambiguous cases, particularly for the clinically nuanced judgments required in ADE identification. Both annotators received training on ADE identification criteria prior to annotation, and a supervising pharmacist (CL) was consulted for cases requiring additional clinical expertise.

During the annotation process, annotators were instructed to make independent determinations about whether an ADE was present based solely on the clinical information documented in the note, regardless of whether the clinician writing the note explicitly suspected or mentioned an ADE. This approach ensured that annotations reflected objective assessment of the clinical documentation rather than simply identifying instances where clinicians had already recognized potential ADEs. For example, if a note documented symptoms consistent with a known medication side effect but the clinician did not explicitly attribute these symptoms to the medication, annotators were still expected to flag this as an ADE if the temporal relationship and clinical presentation supported such a determination.

The classification task was a binary document-level classification: each encounter note received a label of ADE-positive or ADE-negative. During annotation, specific drug names and symptom phrases were marked within the text to guide annotators, but the models were trained and evaluated on the note-level binary label only. The binary classification approach was chosen because (1) the actionable output for pharmacovigilance screening is a note-level flag, (2) NER and RE require substantially more complex annotations with lower agreement rates, and (3) binary classification is more robust to the brevity and abbreviation-heavy nature of primary care notes.

### Model Development and Training

The annotated data were split into a training set (80% of the notes; 868/1085 notes) and a test set (20%; 217/1085 notes), stratified at the note level by class label (ADE-positive vs ADE-negative) using sklearn train_test_split with a fixed random seed for reproducibility. Because the medication-replacement subselection criterion produces a note-level cohort, and our outcome of interest is note-level (whether a given encounter note contains an ADE), splitting at the note level matches the deployment unit of analysis.

The note-level split does not guarantee that every patient appears in only 1 of the 2 splits. Of 998 unique patients in the cohort, 18 (1.8%) have notes in both training and test sets, corresponding to 18 of 217 (8.3%) test notes from “leaked” patients, of which 4 of 71 (5.6%) ADE-positive test notes are from leaked patients. Two patients have ADE-positive notes in both splits, the configuration most permissive of patient-level memorization. To quantify the impact of this overlap on measured performance, we conducted a sensitivity analysis (see “Sensitivity to Patient-Level Leakage” section) reporting both pooled (paired bootstrap across seeds) and per-seed (independent bootstrap per seed) CIs.

All classifiers in the BERT (Bidirectional Encoder Representations from Transformers) family (BlueBERT and BioBERT, both Original and SOAP [Subjective-Objective-Assessment-Plan] variants) were trained with class-weighted CrossEntropy loss using autocomputed weights (weight_neg=1.0, weight_pos=n_neg/n_pos ≈ 2.06) at a learning rate of 2×10^–5^. Each configuration was trained across 5 random seeds (42, 1337, 2024, 31415, and 271828) to enable the reporting of bootstrap CIs on test-set performance.

Models in Table 1 were selected to represent a range of NLP strategies, from fine-tuned encoder transformers (BioBERT and BlueBERT) to a zero-shot LLM classifier (FollowIR) to an embedding-based neural classifier (GritLM+ MLP), in order to compare approaches that differ in their reliance on labeled training data, their use of domain-specific pretraining, and their overall architecture. An additional novel model, the Etiology List Classification pipeline, was developed to mimic clinical reasoning by decomposing the classification task into symptom extraction, differential diagnosis generation, and medication-causation classification. Details on the prompts and hyperparameters used for each model are provided in Multimedia Appendices 1 and 2.

**Table 1.** NLP^a^ models evaluated for ADE^b^ detection in primary care clinical notes.

Model name	Input data	Short description
BioBERT Classifier	Unaltered clinical notes	BioBERT is a BERT^c^-based model pretrained on biomedical abstracts (PubMed and PubMed Central). It was fine-tuned on the labeled training set using the Hugging Face Transformers library with default Training Arguments parameters for binary ADE classification [31,32]. Hyperparameters: Section S3 in Multimedia Appendix 2
BlueBERT Classifier	Unaltered clinical notes	BlueBERT is a BERT-based model pretrained on both biomedical abstracts and clinical discharge summaries (MIMIC-III), making it better suited to clinical language than BioBERT. Fine-tuned on the labeled training set using the same approach as row 1 [33]. Hyperparameters: Section S2 in Multimedia Appendix 2
BlueBERT Classifier	SOAP Rewrite (Mistral-7b-Instruct-v0.1)	Same architecture as row 2, applied to SOAP^d^-rewritten notes (see description above). Hyperparameters: Section S2 in Multimedia Appendix 2
LLM Classifier (FollowIR/Mistral-7b)	Unaltered clinical notes	FollowIR is a prompt-based classification LLM^e^ fine-tuned on Text Retrieval Conference (TREC) datasets, using Mistral-7b as its base model [34,35]. Unlike the BERT-based models, it requires no labeled training data from the target dataset. By simply providing a task description and the encounter note, the model classifies notes as ADE-positive or ADE-negative in a zero-shot setting, responding with a single token (“true” or “false”). Prompt: Section S5 in Multimedia Appendix 1. Hyperparameters: Section S4 in Multimedia Appendix 2
LLM Classifier (FollowIR / Mistral-7b)	SOAP Rewrite (Mistral-7b-Instruct-v0.1)	Same architecture as row 4, applied to SOAP-rewritten notes (see description above). Prompt: Section S5 in Multimedia Appendix 1. Hyperparameters: Section S4 in Multimedia Appendix 2
LLM Embeddings Classifier (GritLM + MLP)	Unaltered clinical notes	GritLM is an LLM that extracts contextual embeddings from clinical notes, mathematical representations of words that capture their meaning in context [36]. These embeddings are passed into a custom 2-layer neural network (a machine learning model that learns patterns through interconnected layers) for binary ADE classification. The GritLM parameters were frozen to prevent overfitting, while the classification layers were trained using ReLU^f^ activation, batch normalization, and dropout. To address class imbalance, a loss multiplier was applied to ADE-containing notes during training, improving precision for the minority class. This architecture balances performance and generalizability, offering a scalable and interpretable approach to ADE detection. Architecture and prompt: Section S6 in Multimedia Appendix 1. Hyperparameters: Section S6 in Multimedia Appendix 2
LLM Embeddings Classifier (GritLM+ MLP)	SOAP Rewrite (Mistral-7b-Instruct-v0.1)	Same architecture as row 6, applied to SOAP-rewritten notes (see description above). Prompt: Section S6 in Multimedia Appendix 1. Hyperparameters: Section S6 in Multimedia Appendix 2
Etiology List Classification (Multistep LLM Pipeline)	SOAP Rewrite Only (Mistral-7b-Instruct-v0.1)	Designed to mimic the decision-making process of a medical professional, this 3-stage pipeline is applied to SOAP-rewritten notes: (1) symptom extraction: Mistral-7b-Instruct-v0.1 extracts individual presenting symptoms from the SOAP-rewritten note. (2) Ranked etiology generation: the extracted symptoms are fed back into Mistral-7b-Instruct-v0.1 along with the corresponding clinical note to retrieve a numbered list of etiologies for each symptom. The model generates these in decreasing order of causal probability, prioritizing the treating clinician’s implicit or explicit views. (3) Binary classification via FollowIR: the top 2 etiologies from each ranked list are passed to FollowIR for medication-causation classification. Note-level aggregation uses OR logic; if any symptom within a note is classified as medication-caused (score>0.5), the entire note is labeled ADE-positive. This prioritizes recall, consistent with a pharmacovigilance screening objective. Prompts: Sections S1 (SOAP rewrite), S2 (symptom extraction), S3 (etiology ranking), and S4 (FollowIR classification) in Multimedia Appendix 1. Hyperparameters: Sections S4 (FollowIR) and S5 (Mistral-7b) in Multimedia Appendix 2

a NLP: natural language processing.

b ADE: adverse drug event.

c BERT: Bidirectional Encoder Representations from Transformers.

d SOAP: Subjective-Objective-Assessment-Plan.

e LLM: large language model.

f ReLU: rectified linear unit.

Each model was evaluated on unaltered clinical notes. A subset was additionally evaluated on notes rewritten into SOAP format using Mistral-7b-Instruct-v0.1, and fine-tuned models were retrained on the rewritten notes, while the zero-shot LLM Classifier was reevaluated without additional training. Providers often use shorthand terminology and abbreviations, and clinical notes frequently lack structure [34]. SOAP notes help ensure thorough, organized documentation and make it easier for other health care providers to quickly understand a patient’s situation. They are used by doctors, nurses, therapists, and other health care professionals across various settings [37]. It was hypothesized that rewriting notes into SOAP format, which ensures thorough, organized documentation used widely across health care settings, would improve classifier performance.

FollowIR-7B is a Mistral-7B model fine-tuned on the FollowIR training set, which adapts narrative-style instructions originally developed for human assessors at the Text Retrieval Conference (TREC) to teach Information Retrieval (IR) models to follow detailed natural-language relevance instructions. The capability acquired during this fine-tuning is general, produces a binary relevance judgment in response to an instruction-document pair, and transfers naturally to our task: given an instruction describing what constitutes an ADE, it classifies whether a clinical note (or, in the etiology pipeline, a [drug and symptom] candidate) instantiates one. We use this capability zero-shot, with no additional fine-tuning. The Mistral-7B base provides baseline language understanding through pretraining on a broad corpus that includes biomedical text, though FollowIR’s specific fine-tuning is on general (nonbiomedical) TREC collections. We include FollowIR as a label-free LLM baseline complementing our supervised classifiers, not as a primary clinical reasoning engine; limitations of this framing are discussed below.

For models trained using notes rewritten in SOAP format, the following safeguards against label leakage and hallucination were implemented:

1. ADE labels were not provided to Mistral-7b during rewriting. The model received only the raw encounter note text.
2. The rewriting prompt contained no references to ADEs or any classification objective. The prompt was: “Rewrite the provided encounter note in Subjective-Objective-Assessment-Plan (SOAP) format. Reorganize the existing content into SOAP structure without adding any information that is not explicitly stated in the note. Do not infer diagnoses, causes, severity, or clinical reasoning that the note does not state. Expand abbreviations and shorthand to standard medical equivalents only when the meaning is unambiguous; if a term is ambiguous, leave it as written. Limit yourself strictly to the information in the note.”
3. The prompt explicitly prohibits inference of diagnoses, causes, severity, or clinical reasoning, constraining rewriting to structural reorganization and unambiguous abbreviation expansion.

To validate information fidelity, we conducted a structured qualitative review of 12 notes and their corresponding SOAP rewrites. The rewrites limited themselves to the information in the encounter notes and expanded shorthand and abbreviations; no fabricated entities (drugs, symptoms, dates, or values) were observed. This audit, the revised prompt above, and the per-seed regeneration that produces 5 independent SOAP datasets together constitute the validation we performed; an exhaustive larger-model comparison and a systematic LLM-as-judge evaluation are reserved for future work.

### Model Performance Assessment

Model performance was evaluated using 3 standard metrics commonly used in binary classification tasks: precision, recall, and *F*_1_-score. These metrics were calculated based on the model’s ability to correctly identify encounter notes containing ADEs. Precision (positive predictive value [PPV]) measures the proportion of encounter notes predicted to contain ADEs that contained ADEs (true positives/(true positives + false positives)). High precision is critical in ADE detection to minimize false alarms that could lead to unnecessary clinical review or inappropriate medication changes. A model with low precision would flag many encounters as containing ADEs when they do not, potentially overwhelming clinicians with false alerts.

Recall (sensitivity) measures the proportion of actual ADE-containing notes that were correctly identified by the model (true positives/(true positives + false negatives)). High recall ensures that potentially serious ADEs are not missed. In pharmacovigilance, failing to detect an ADE could result in continued patient exposure to harmful medications, making recall a crucial safety metric.

*F*_1_-score represents the harmonic mean of precision and recall (2 × (precision × recall)/(precision + recall)), providing a balanced measure of model performance. This metric is particularly valuable when dealing with imbalanced datasets, as is typical in ADE detection where ADE-containing notes represent a minority class (355 of 1085 notes, or 32.7% in our dataset). The *F*_1_-score penalizes models that achieve high performance in one metric at the expense of the other.

We also report 2 threshold-independent metrics in Table 2 and Multimedia Appendix 3. Unlike precision, recall, and *F*_1_-score, these do not depend on a single decision threshold. ROC-AUC is the receiver operating characteristic area under the curve of the true positive rate (recall) against the false positive rate, as the threshold is varied. It tells us how well the model ranks ADE-positive notes above ADE-negative ones. It can be read as the probability that a randomly chosen ADE-positive note scores higher than a randomly chosen ADE-negative note. A value of 0.5 is no better than chance and 1.0 is a perfect ranking. PR-AUC is the precision-recall area under the curve. For an imbalanced dataset, such as ours, PR-AUC is the more informative of the two, because it looks at performance on the minority (ADE-positive) class and is not inflated by the large number of true negatives. A model with no predictive ability would score a PR-AUC equal to the prevalence of the positive class, which is 0.327 here, so PR-AUC should be interpreted relative to that floor of 0.327 rather than to zero, with higher values indicating better-than-chance ranking of the minority (ADE-positive) class.

To support multiseed evaluation, all models were trained and evaluated independently across 5 random seeds (42, 1337, 2024, 31415, and 271828). For each model and metric, we report per-seed point estimates with bootstrap 95% CIs (test-set resampling with model fixed; n_bootstrap=1000 per seed) and pooled bootstrap 95% CIs (paired bootstrap across seeds, same resample applied to all 5 seeds within each iteration; n_bootstrap×5 seeds=5000 metric values pooled).

We characterized the input length distribution to confirm fit within the BERT 512-token limit. Token counts were computed using the BlueBERT WordPiece tokenizer (the BioBERT vocabulary is shared and yields equivalent counts within 1‐2 tokens). Original encounter notes have a median length of 107 (IQR 61-156, max 946) tokens, with 9 of 1085 (0.8%) notes exceeding 512 tokens; these were truncated. SOAP-rewritten notes have a median length of 403 (IQR 311-523, max 1505) tokens, with 286 of 1085 (26.4%) notes exceeding 512 tokens. The 512-token limit is fixed by the BERT-base architecture (max position embeddings=512); GritLM (32 k context) and FollowIR (32 k context, Mistral-7B backbone) accept all inputs in full.

**Table 2.** Performance metrics of natural language processing models for detection of clinical notes containing adverse drug events^a^.

Model	Mean *F*_1_-score (%; 95% CI)	Precision (95% CI)	Recall (95% CI)	ROC-AUC^b^ (95% CI)	PR-AUC^c^ (95% CI)
GritLM-SOAP	72.53 (62.40-81.82)	71.55 (56.41-83.93)	74.08 (60.56-88.89)	0.849 (0.779-0.920)	0.764 (0.646-0.870)
GritLM-Original	70.25 (60.74-78.26)	73.21 (60.41-85.71)	67.89 (54.43-80.28)	0.827 (0.757-0.893)	0.758 (0.657-0.847)
BioBERT-SOAP weighted	69.54 (55.29-82.50)	64.03 (46.88-79.17)	76.34 (62.69-89.66)	0.846 (0.742-0.934)	0.750 (0.618-0.898)
BlueBERT-SOAP weighted	67.51 (47.79-80.27)	68.92 (55.00-83.08)	67.32 (39.68-86.96)	0.856 (0.754-0.925)	0.750 (0.606-0.877)
BlueBERT-Original weighted	64.35 (46.81-77.71)	64.24 (44.44-80.56)	64.51 (47.22-78.75)	0.819 (0.699-0.909)	0.733 (0.531-0.874)
FollowIR-SOAP	63.75 (52.38-73.42)	55.53 (43.75-67.50)	74.93 (62.02-86.11)	0.811 (0.740-0.875)	0.666 (0.555-0.777)
FollowIR-Original	61.71 (52.56-70.21)	51.92 (42.42-62.11)	76.06 (65.79-85.71)	0.821 (0.763-0.877)	0.675 (0.565-0.784)
Etiology pipeline	60.29 (47.69-70.50)	62.88 (49.21-75.81)	58.03 (43.59-71.23)	0.742 (0.634-0.845)	0.631 (0.485-0.763)
BioBERT-Original weighted	59.78 (42.75-72.61)	57.60 (42.42-74.55)	62.54 (40.91-79.49)	0.792 (0.683-0.879)	0.647 (0.519-0.780)

a Reported as mean across 5 random seeds with 95% bootstrap CIs (paired bootstrap; n_bootstrap×5 seeds=5000 metric values pooled; n_test=217 notes, n_positive=71). *F*_1_-score, precision, and recall as percentages; AUC values in [0,1].

b ROC-AUC: receiver operating characteristic area under the curve.

c PR-AUC: precision-recall area under the curve.

### NER and RE

Beyond document-level classification of whether an encounter note contains an ADE, we extended the pipeline to span-level extraction: identifying the specific drug and symptom entity mentions and the relations between them. We trained NER and RE components and evaluated each individually and in combination as an end-to-end pipeline.

For NER, we used all 868 notes in the training set (both ADE-positive and ADE-negative). ADE-negative notes contribute as sequences containing no drug or symptom entities; their inclusion teaches the model what mentions are absent in negative contexts. We used BIO tagging with 2 entity types (drug and symptom).

For RE, we used only the 284 ADE-positive notes in the training set. Within each, every (drug and symptom) entity pair becomes a labeled instance: positive if the pair appears as an ADE-drug relation in the BRAT annotation file, negative otherwise. ADE-negative notes contain no relations and would contribute only trivial empty-relation sets to the dataset; we excluded them.

We compared BlueBERT and BioBERT as backbones for both NER and RE, training each over the same 5 seeds used for binary classification and reporting per-seed performance with bootstrap 95% CIs (n_bootstrap=1000 per seed; resampling at the note level for NER and the entity-pair level for RE). NER used a token classification head over the BERT backbone (learning rate 5 × 10^–5^, 5 epochs, batch size 8, AdamW optimizer, and max sequence length 512). RE used a pair classification head: drug and symptom entity spans within a note were marked with special entity-marker tokens, and the BERT-encoded representation of the marked input was passed to a classification head that scored the pair as ADE-drug or not (learning rate 1e-5, 5 epochs, batch size 16, AdamW with linear warmup over the first 10% of training steps, class-weighted loss with pos_weight=sqrt(n_neg/n_pos)≈3.74, and max sequence length 512).

The end-to-end pipeline composes NER and RE sequentially. For each test note, NER predicts drug and symptom spans; we then enumerate all (drug and symptom) pairs and pass each to RE for ADE-drug classification. The output is the set of predicted ADE-drug pairs per note. We compare predicted pairs against gold ADE-drug relations from the BRAT annotation files using 2 evaluation modes: strict (exact span boundary match) and relaxed (token-level overlap between predicted and gold spans). Pipeline evaluation is restricted to the 71 ADE-positive notes in the test set, where at least one gold relation exists.

### Ethical Considerations

Ethics approval was granted by the University of Manitoba’s Health Research Ethics Board (HS20263 H2016:408). The study protocol was reviewed by the Manitoba Provincial Health Research Privacy Committee (PHRPC Number P2023-22). All data in the Repository are deidentified prior to inclusion. All models, including Mistral-7b-Instruct and FollowIR, were run locally on secure institutional hardware within the air-gapped research environment. No clinical data were sent to any external or cloud-based service at any stage, consistent with the approved data governance and data sharing agreements.

## Results

### Overview

The annotators identified 355 encounter notes with ADEs, out of a total of 1085 annotated notes. The detailed breakdown of the encounter notes, as found during 2 sequential rounds of the annotation process, using encounter notes drawn from the same data source, is shown in Table 3.

Table 4 summarizes the patient-level ADE metrics, including the number of unique patients represented in the clinical notes, those with at least one ADE, those with multiple ADEs, and the average number of ADEs per patient.

Table 5 presents a breakdown of patient sex and age. Sex and age are both reported at the patient level (denominator=998 unique patients), since sex is invariant per patient and we use each patient’s age at their first note as a single representative value. The note-level rate of ADE-positive notes by patient sex is reported in a footnote.

**Table 3.** Summary of primary care clinical notes identified as ADE^a^-positive during the annotation process.

Annotation wave	Total notes	Notes containing ADEs
Wave 1	258	68
Wave 2	827	287
Total	1085	355

a ADE: adverse drug event.

**Table 4.** Summary of patient-level ADE^c^ metrics as identified during the annotation process.

Patient-level metric	Count (%)
Total unique patients	998 (100)
Patients with at least 1 ADE	347 (34.8)
Patients with multiple ADEs	7 (0.7)
Average ADEs per patient	1.02^b^

a ADE: adverse drug event.

b Mean number of adverse drug events among the 347 patients with at least one adverse drug event. This value is a mean, not a count or proportion.

**Table 5.** Patient demographics of the cohort (n=998 unique patients across 1085 notes).

Patient characteristic	Value, n (%)
Sex	
Male	447 (44.8)
Female	551 (55.2)
Age at first note**^a^** (years)	
<55	0 (0)
55‐59	5 (0.5)
60‐64	159 (15.9)
65‐69	204 (20.4)
70‐74	218 (21.8)
75‐79	148 (14.8)
80‐84	135 (13.5)
≥85	129 (12.9)

a Sex and age are reported at the patient level, since sex is invariant per patient and age is taken at the patient’s first note. The note-level rate of adverse drug event–positive notes by patient sex was 28.6% among male patients (136 of 475 notes) and 35.9% among female patients (219 of 610 notes).

Tables 6 and 7 decompose the cohort into the training and test splits used for model development and evaluation, at the patient level and the note level, respectively. Of the 998 unique patients, 802 contributed at least one training note and 214 contributed at least one test note; 18 patients (12 females and 6 males) appear in both splits, so the row totals across splits exceed 998 by 18. The split itself was originally generated by stratified random sampling on the binary ADE label at the note level, which is why a small number of patients with multiple notes have notes in both splits. The implications of this patient-level leakage for model evaluation are addressed in the subsection “Sensitivity to Patient-Level Leakage” under the “Results” section. Percentages in the “Training” and “Test” columns are shares of the column total; percentages in the “Training ADE+” and “Test ADE+” columns are row-wise rates within each sex or age band (eg, 108 of 355 male training patients=30.4%).

The performance metrics of the models over the test set, evaluated across 5 random seeds with bootstrap 95% CIs, are presented in Table 2 above. SOAP rewriting generally improved performance for the BERT-family classifiers and the LLM Embeddings Classifier (eg, GritLM mean *F*_1_-score increased from 70.25% on original to 72.53% on SOAP; BlueBERT-weighted mean *F*_1_-score increased from 64.35% to 67.51%) while having little effect on the FollowIR classifier (mean *F*_1_-score 61.71% on Original to 63.75% on SOAP).

Per-seed point estimates and per-seed bootstrap CIs (test-set resampling with model fixed) are provided in the Multimedia Appendix 3.

**Table 6.** Patient-level distribution by training and test split^a^.

Patient characteristic	Training (n=802)	Training ADE+^b^ (n=279)	Test (n=214)	Test ADE+ (n=70)
Sex, n (%)				
Male	355 (44.3)	108 (30.4)	98 (45.8)	27 (27.6)
Female	447 (55.7)	171 (38.3)	116 (54.2)	43 (37.1)
Age (at first note; years), n (%)				
55‐59	4 (0.5)	0 (0.0)	1 (0.5)	0 (0.0)
60‐64	127 (15.8)	50 (39.4)	33 (15.4)	12 (36.4)
65‐69	169 (21.1)	58 (34.3)	39 (18.2)	15 (38.5)
70‐74	166 (20.7)	71 (42.8)	55 (25.7)	18 (32.7)
75‐79	122 (15.2)	42 (34.4)	30 (14.0)	12 (40.0)
80‐84	120 (15.0)	34 (28.3)	18 (8.4)	5 (27.8)
≥85	94 (11.7)	24 (25.5)	38 (17.8)	8 (21.1)

a Each patient counted once per split; ADE+ columns show patients with≥1 adverse drug event–positive note in that split.

b ADE: adverse drug event.

**Table 7.** Note-level distribution by training and test split^a^.

Patient characteristic	Training (n=868)	Training ADE+^b^ (n=284)	Test (n=217)	Test ADE+ (n=71)
Sex (of the patient associated with the note), n (%)				
Male	375 (43.2)	109 (29.1)	100 (46.1)	27 (27)
Female	493 (56.8)	175 (35.5)	117 (53.9)	44 (37.6)
Age (at the note’s encounter date; years), n (%)				
55‐59	4 (0.5)	0 (0)	1 (0.5)	0 (0)
60‐64	133 (15.3)	49 (36.8)	33 (15.2)	12 (36.4)
65‐69	193 (22.2)	60 (31.1)	39 (18.0)	15 (38.5)
70‐74	177 (20.4)	73 (41.2)	55 (25.3)	18 (32.7)
75‐79	131 (15.1)	42 (32.1)	32 (14.7)	13 (40.6)
80‐84	124 (14.3)	35 (28.2)	19 (8.8)	5 (26.3)
≥85	106 (12.2)	25 (23.6)	38 (17.5)	8 (21.1)

a Each note counted once; ADE+ columns show adverse drug event–positive notes within that split (denominator is column total).

b ADE: adverse drug event.

### Span-Level Extraction Performance

Tables 8 and 9 report per-seed NER metrics for BlueBERT and BioBERT, respectively, with 95% bootstrap CIs on the test-set sample (model fixed within each row). BlueBERT outperformed BioBERT on every metric and every seed, and per-seed CIs do not overlap on any metric.

The substantial gap between strict and relaxed scores (eg, BlueBERT seed 42: 0.524 to >0.758) indicates that the model frequently identifies entities at approximately the right location but with imperfect span boundaries, a known characteristic of token-classification NER on clinical text where annotation conventions for span boundaries vary. We selected BlueBERT-NER for use in the end-to-end pipeline.

Tables 10 and 11 report per-seed RE metrics with 95% bootstrap CIs on the test-pair sample. BioBERT outperformed BlueBERT on every metric and every seed, the opposite ordering to NER.

**Table 8.** BlueBERT-NER per-seed performance^a^.

Seed	Strict *F*_1_-score (95% CI)	Relaxed *F*_1_-score (95% CI)	Strict drug *F*_1_-score (95% CI)	Strict symptom *F*_1_-score (95% CI)	Relaxed drug *F*_1_-score (95% CI)	Relaxed symptom *F*_1_-score (95% CI)
42	0.524 (0.489-0.558)	0.758 (0.716-0.791)	0.734 (0.693-0.775)	0.380 (0.336-0.423)	0.886 (0.867-0.904)	0.672 (0.616-0.719)
1337	0.503 (0.463-0.537)	0.738 (0.687-0.775)	0.720 (0.674-0.762)	0.366 (0.322-0.406)	0.885 (0.864-0.904)	0.647 (0.577-0.701)
2024	0.497 (0.452-0.535)	0.717 (0.658-0.759)	0.729 (0.685-0.770)	0.361 (0.314-0.405)	0.888 (0.867-0.906)	0.620 (0.548-0.677)
31415	0.481 (0.440-0.523)	0.698 (0.637-0.745)	0.714 (0.670-0.753)	0.347 (0.302-0.392)	0.870 (0.850-0.891)	0.600 (0.522-0.663)
271828	0.499 (0.460-0.537)	0.734 (0.688-0.772)	0.745 (0.705-0.783)	0.355 (0.312-0.398)	0.893 (0.875-0.910)	0.645 (0.585-0.697)
Mean (SD)	0.500 (0.016)	0.728 (0.023)	0.729 (0.013)	0.361 (0.014)	0.884 (0.009)	0.637 (0.029)

a *F*_1_-score values are decimals.

**Table 9.** BioBERT-NER per-seed performance^a^.

Seed	Strict *F*_1_-score (95% CI)	Relaxed *F*_1_-score (95% CI)	Strict drug *F*_1_-score (95% CI)	Strict symptom *F*_1_-score (95% CI)	Relaxed drug *F*_1_-score (95% CI)	Relaxed symptom *F*_1_-score (95% CI)
42	0.222 (0.200-0.242)	0.514 (0.478-0.545)	0.361 (0.326-0.395)	0.140 (0.121-0.159)	0.609 (0.569-0.644)	0.461 (0.420-0.498)
1337	0.271 (0.247-0.294)	0.575 (0.539-0.608)	0.471 (0.429-0.511)	0.164 (0.141-0.187)	0.732 (0.701-0.760)	0.493 (0.452-0.531)
2024	0.188 (0.169-0.206)	0.491 (0.457-0.523)	0.360 (0.326-0.393)	0.114 (0.098-0.131)	0.670 (0.640-0.698)	0.418 (0.378-0.458)
31415	0.195 (0.175-0.217)	0.467 (0.427-0.504)	0.362 (0.325-0.401)	0.117 (0.099-0.136)	0.630 (0.600-0.660)	0.393 (0.347-0.439)
271828	0.184 (0.164-0.205)	0.478 (0.440-0.513)	0.334 (0.301-0.367)	0.112 (0.096-0.130)	0.604 (0.575-0.634)	0.420 (0.376-0.464)
Mean (SD)	0.212 (0.036)	0.505 (0.043)	0.378 (0.053)	0.129 (0.023)	0.649 (0.053)	0.437 (0.040)

a *F*_1_-score values are decimals.

**Table 10.** BlueBERT-RE per-seed performance^a^.

Seed	*F*_1_-score (95% CI)	Precision (95% CI)	Recall (95% CI)	ROC-AUC^b^ (95% CI)	PR-AUC^c^ (95% CI)
42	0.542 (0.450-0.626)	0.585 (0.474-0.683)	0.507 (0.410-0.608)	0.888 (0.849-0.926)	0.493 (0.385-0.594)
1337	0.440 (0.347-0.528)	0.467 (0.359-0.565)	0.419 (0.323-0.524)	0.794 (0.738-0.843)	0.367 (0.268-0.465)
2024	0.422 (0.329-0.509)	0.428 (0.333-0.528)	0.420 (0.321-0.517)	0.864 (0.826-0.897)	0.400 (0.301-0.497)
31415	0.478 (0.396-0.560)	0.429 (0.336-0.519)	0.543 (0.444-0.644)	0.870 (0.830-0.906)	0.450 (0.343-0.559)
271828	0.526 (0.434-0.609)	0.558 (0.450-0.663)	0.499 (0.398-0.598)	0.908 (0.874-0.937)	0.527 (0.414-0.628)
Mean (SD)	0.483 (0.053)	0.493 (0.075)	0.478 (0.056)	0.865 (0.043)	0.441 (0.067)

a *F*_1_-score, precision, and recall as decimals; AUC values in [0,1].

b ROC-AUC: receiver operating characteristic area under the curve.

c PR-AUC: precision-recall area under the curve.

**Table 11.** BioBERT-RE per-seed performance^a^.

Seed	*F*_1_-score (95% CI)	Precision (95% CI)	Recall (95% CI)	ROC-AUC^b^ (95% CI)	PR-AUC^c^ (95% CI)
42	0.603 (0.521-0.675)	0.594 (0.487-0.696)	0.612 (0.514-0.706)	0.930 (0.901-0.957)	0.596 (0.486-0.700)
1337	0.563 (0.479-0.639)	0.489 (0.402-0.581)	0.663 (0.564-0.755)	0.926 (0.901-0.948)	0.564 (0.456-0.667)
2024	0.578 (0.498-0.659)	0.557 (0.456-0.654)	0.602 (0.503-0.702)	0.931 (0.906-0.952)	0.631 (0.527-0.731)
31415	0.555 (0.469-0.630)	0.488 (0.391-0.578)	0.643 (0.541-0.737)	0.943 (0.918-0.964)	0.626 (0.518-0.728)
271828	0.567 (0.477-0.642)	0.489 (0.397-0.583)	0.673 (0.578-0.769)	0.940 (0.917-0.961)	0.620 (0.500-0.731)
Mean (SD)	0.573 (0.019)	0.523 (0.049)	0.639 (0.031)	0.934 (0.007)	0.607 (0.028)

a *F*_1_-score, precision, and recall as decimals; AUC values in [0,1].

b ROC-AUC: receiver operating characteristic area under the curve.

c PR-AUC: precision-recall area under the curve.

The BlueBERT-RE and BioBERT-RE *F*_1_-score CIs overlap on individual seeds. AUC (area under the curve) intervals do not overlap on 3 of 5 seeds, and BioBERT-RE mean AUC exceeds BlueBERT-RE mean AUC on every seed, supporting BioBERT-RE selection. We selected BioBERT-RE for use in the end-to-end pipeline.

Table 12 reports per-seed end-to-end performance on the 71 ADE-positive test notes, with 95% bootstrap CIs on the test-set sample (n_bootstrap=1000 per seed; model fixed within each row).

Because this evaluation is restricted to the 71 ADE-positive notes, these are oracle metrics. They represent an idealized 2-stage pipeline (NER+ RE) in which all ADE-negative notes have been perfectly filtered out before extraction, so they should not be read as deployment performance. The deployment-realistic 3-stage cascade, in which a binary classifier first gates extraction and imperfectly filters negatives, is reported in the “Discussion” section, with full per-seed results in Section S4 in Multimedia Appendix 3.

Per-seed performance varied meaningfully; strict *F*_1_-score ranged from 0.293 (seed 271828) to 0.409 (seed 42), reflecting the small test set size and accumulated stochasticity from training NER and RE under different seeds. Seed 42 yielded the strongest pipeline.

To diagnose where the end-to-end pipeline fails, we attributed each false negative (a missed gold ADE-drug pair) to 1 of 4 categories: (1) NER missed the drug span, (2) NER missed the symptom span, (3) NER missed both spans, or (4) NER produced both spans correctly but RE failed to classify them as an ADE-drug pair. The counts and proportions, summed across 5 seeds and reported separately for strict and relaxed evaluation, are in Table 13.

At relaxed-span matching, NER is essentially solved on this dataset; the bottleneck is RE. Improving RE (rather than entity recognition) would yield the largest gains for end-to-end performance.

**Table 12.** End-to-end pipeline (BlueBERT-NER+ BioBERT-RE) on ADE^a^-positive test notes (n=71)^b^.

Seed	Strict *F*_1_-score (95% CI)	Strict precision (95% CI)	Strict recall (95% CI)	Relaxed *F*_1_-score (95% CI)	Relaxed precision (95% CI)	Relaxed recall (95% CI)
42	0.409 (0.321-0.502)	0.362 (0.277-0.452)	0.469 (0.359-0.586)	0.533 (0.449-0.615)	0.472 (0.390-0.558)	0.612 (0.490-0.726)
1337	0.322 (0.243-0.400)	0.245 (0.184-0.314)	0.469 (0.360-0.586)	0.448 (0.372-0.521)	0.340 (0.278-0.409)	0.653 (0.529-0.774)
2024	0.368 (0.287-0.455)	0.312 (0.239-0.397)	0.449 (0.350-0.556)	0.469 (0.386-0.545)	0.397 (0.322-0.483)	0.571 (0.459-0.673)
31415	0.298 (0.232-0.368)	0.223 (0.172-0.283)	0.449 (0.343-0.562)	0.407 (0.335-0.480)	0.305 (0.245-0.373)	0.612 (0.495-0.731)
271828	0.293 (0.221-0.370)	0.218 (0.162-0.281)	0.449 (0.330-0.566)	0.420 (0.356-0.489)	0.312 (0.259-0.374)	0.643 (0.532-0.760)
Mean (SD)	0.338 (0.049)	0.272 (0.063)	0.457 (0.011)	0.455 (0.050)	0.365 (0.070)	0.618 (0.032)

a ADE: adverse drug event.

b Per-seed point estimates with 95% bootstrap CIs on the test set.

**Table 13.** Error attribution among end-to-end false negatives, summed across 5 seeds.

Mode	Total FN^a^	NER^b^-drug, n (%)	NER-symptom, n (%)	NER-both, n (%)	RE^c^-pair, n (%)
Strict	266	24 (9)	117 (44)	11 (4)	114 (43)
Relaxed	187	0 (0)	7 (4)	0 (0)	180 (96)

a FN: false negative.

b NER: named entity recognition.

c RE: relation extraction.

### Sensitivity to Patient-Level Leakage

Of 217 test notes, 18 (8.3%) come from patients who also have notes in the training set. Excluding these leaked notes leaves a clean subset of 199 test notes (67 ADE-positive and 132 ADE-negative). We reevaluated all 9 models on this clean subset across 5 seeds and report 2 complementary views: a pooled view (Table 14) and a per-seed view in Multimedia Appendix 3.

At the population level (mean across 5 seeds with paired bootstrap CIs), Δ *F*_1_-score ranges from +0.53 to +1.95 percentage points across the model panel (all positive), and Δ ROC-AUC ranges from −0.001 to +0.016. Both ranges sit comfortably within the per-model bootstrap CIs.

**Table 14.** Pooled sensitivity of model performance to exclusion of patient-leaked test notes^a^.

Model	Full *F*_1_-score (%; 95% CI)	Clean *F*_1_-score (95% CI)	Δ *F*_1_-score	Full AUC^b^ (95% CI)	Clean AUC (95% CI)	Δ AUC
GritLM-SOAP	72.53 (62.40-81.82)	74.48 (63.38-84.34)	+1.95	0.849 (0.779-0.920)	0.858 (0.784-0.935)	+0.009
GritLM-Original	70.25 (60.74-78.26)	71.09 (60.80-79.63)	+0.84	0.827 (0.757-0.893)	0.831 (0.755-0.903)	+0.004
BioBERT-SOAP weighted	69.54 (55.29-82.50)	70.28 (56.00-83.44)	+0.74	0.846 (0.742-0.934)	0.852 (0.750-0.941)	+0.006
BlueBERT-SOAP weighted	67.51 (47.79-80.27)	68.82 (48.07-81.63)	+1.30	0.856 (0.754-0.925)	0.862 (0.760-0.932)	+0.006
BlueBERT-Original weighted	64.35 (46.81-77.71)	64.89 (46.15-78.83)	+0.53	0.819 (0.699-0.909)	0.819 (0.691-0.913)	−0.001
BioBERT-Original weighted	59.78 (42.75-72.61)	61.50 (43.75-74.39)	+1.72	0.792 (0.683-0.879)	0.804 (0.684-0.893)	+0.012
FollowIR-SOAP	63.75 (52.38-73.42)	65.24 (53.06-75.18)	+1.49	0.811 (0.740-0.875)	0.819 (0.742-0.885)	+0.008
FollowIR-Original	61.71 (52.56-70.21)	63.35 (54.32-71.17)	+1.64	0.821 (0.763-0.877)	0.837 (0.781-0.892)	+0.016
Etiology pipeline	60.29 (47.69-70.50)	61.20 (48.69-72.13)	+0.92	0.742 (0.634-0.845)	0.751 (0.643-0.853)	+0.010

a Full *F*_1_-score and AUC are mean across 5 seeds on the full 217-note test set with paired bootstrap 95% CIs (matching Table 2). Clean *F*_1_-score and AUC are the mean across 5 seeds on the 199-note clean subset (the 18 patient-leaked notes excluded) with paired bootstrap 95% CIs. Δ values (clean minus full) are point estimates of the difference in means; the full and clean CIs are computed from independent bootstrap runs over different sample spaces (217 vs 199 notes), so CI overlap should be interpreted as describing precision around each estimate rather than as a paired hypothesis test. *F*_1_-score as percentages; AUC values in [0,1].

b AUC: area under the curve.

The supplementary per-seed tables in Multimedia Appendix 3 (one per model) report individual seeds’ clean *F*_1_-score and AUC with independent bootstrap CIs. Per-seed Δ values vary substantially within models (ranges of 11‐22 percentage points in *F*_1_-score between best and worst seed for the BERT-family models). This intramodel variability is consistent with random test-set sampling rather than systematic patient-level memorization: were models exploiting patient-specific features, leaked notes would be systematically easier across all seeds, producing uniformly negative Δ values. Instead, every model has both positive and negative per-seed Δ values, and the direction is unrelated to the leakage status of the excluded notes; different seeds happen to perform differently on the leaked subset versus the rest of the test set, with no consistent bias.

We did not stratify test-set performance by single-encounter versus multiencounter patients because the test set contains 214 unique patients across 217 notes, with only 3 multiencounter patients in the test set, with 6 notes between them. This sample size precludes a meaningful subgroup comparison. The sensitivity analysis above addresses the underlying concern (whether the model is exploiting patient-level recurrence) using all available data.

## Discussion

### Study Design

In this study, we aimed to create ADE classifiers tailored for single-patient visit clinical notes—a task that has not been undertaken by the major publicly available datasets and challenges. The n2c2 and MADE 1.0 challenges used detailed discharge summaries, and the latter only focused on encounter notes of 21 patients with a cancer diagnosis [6,9]. In comparison, our dataset was drawn from a wide variety of primary care clinical notes, a more random and representative sample of ADE-containing clinical notes.

Our study’s primary focus was note-level binary classification, with span-level extraction (NER+RE) developed as a complementary downstream component. The reasons for prioritizing the binary classification task were 3-fold. First, our intended use case is pharmacovigilance screening, flagging notes for human review, where a binary signal is sufficient to route notes to clinical experts who can then identify specific drug-ADE pairs. Second, full NER and RE require annotating all drug and symptom mentions across all notes regardless of ADE status, which represents a substantially more costly annotation effort. Third, primary care notes were found to often be brief and use extensive abbreviations, making reliable span-level annotation particularly challenging.

We extended the analysis to include end-to-end span-level extraction (see “Span-Level Extraction Performance” section). Drug and symptom spans were available for ADE-positive documents in the annotation set, which made this extension feasible without further annotation effort. The headline finding from that analysis is that, at relaxed-span matching, NER on this dataset is essentially solved while the bottleneck for end-to-end span extraction is RE; this directs subsequent improvement work to a specific component of the pipeline.

The decision to allow annotators to identify ADEs not explicitly suspected by clinicians was intentional, reflecting our goal of detecting underreported ADEs—a key study motivation. Literature estimates that up to 90% of ADEs go unreported in structured data fields [20]. Restricting annotations to clinician-suspected ADEs would have reproduced the very gap our system aims to address. To mitigate subjectivity: (1) annotators were pharmacy students with clinical knowledge of drug-symptom relationships, (2) annotations required temporal plausibility between drug administration and symptom onset, and (3) a supervising pharmacist was consulted for ambiguous cases.

### Principal Findings

Models trained on clinical notes rewritten in SOAP format performed better than the same model trained on the original, unaltered clinical notes (Table 2). This could be due to various factors. First, the SOAP restructuring process forces the LLM to expand abbreviated terminology and medical shorthand into complete terms, making the text more accessible to downstream models. Second, the SOAP format explicitly separates subjective symptoms from objective findings and clinical assessments, which may help models better distinguish between patient-reported experiences (where ADEs often appear) and other clinical information. Finally, the standardization process may reduce noise from inconsistent documentation styles across different providers, allowing models to focus on clinically relevant content rather than stylistic variations.

Models were compared primarily using *F*_1_-score to identify those achieving the best balance, while precision and recall provided more granular performance measures that allow researchers to select models based on the use case. For instance, BioBERT-SOAP weighted achieved the highest mean recall in Table 2 (76.34%) and could be used as a screening tool where reducing missed ADEs is the priority, while GritLM-Original achieved the highest mean precision (73.21%) and could be used in a clinical decision support system that requires higher precision to maintain clinician confidence. GritLM-SOAP achieved the strongest mean *F*_1_-score (72.53%, SD 3.10) with relatively low seed-to-seed variability, making it a balanced default for general use.

The early findings of zero performance of BioBERT (when trained without class weighting) reflected the model collapsing to the majority (non-ADE) class for all test instances. With class weighting applied during fine-tuning (weight_pos=n_neg / n_pos ≈ 2.06), all 4 BERT-family configurations now produce nontrivial classifiers (Table 2). However, BioBERT-Original-weighted shows substantial seed-to-seed variability (per-seed *F*_1_-score mean 59.78%, SD 7.06; range 48.92‐66.67 across seeds). This stability finding suggests that this configuration sits closer to the edge of training stability on a small dataset; researchers reproducing this work should expect comparable variability.

BlueBERT’s superior performance on the same configuration likely reflects its additional pretraining on clinical text (MIMIC-III discharge summaries), providing more relevant representations for clinical note classification.

The end-to-end span-level pipeline (see “Span-Level Extraction Performance” section) achieved a mean relaxed *F*_1_-score of 0.455 across 5 seeds, well below the document-classifier mean *F*_1_-score of 72.53% (SD 3.10) from GritLM-SOAP. Two factors contribute to this gap. First, the span-level task is substantially harder than the document-level task: it requires identifying not only that a note contains an ADE but also which specific drug-symptom pair instantiates the ADE, with correct span boundaries (or at least span overlap, in the relaxed case). Second, a multistage pipeline necessarily compounds errors: a drug or symptom span missed by NER can never be recovered by RE, and a correctly identified pair can still be misclassified by RE. The error-attribution analysis (Table 13) showed that, at relaxed-span matching on this dataset, the dominant failure mode is the latter: 96% of relaxed false negatives are pairs that NER detected correctly but RE failed to link as ADE-drug. This suggests that future work to improve end-to-end span-level performance should prioritize RE modeling (potentially via richer pair representations, larger annotated training sets, or more sophisticated relation-classification objectives) rather than further refinement of NER components.

A deployment-realistic 3-stage cascade, GritLM-SOAP binary classifier gating BlueBERT-NER and BioBERT-RE, was also evaluated on all 217 test notes, with notes classified as ADE-negative by the binary stage contributing zero predicted relations (their gold ADE-drug pairs become false negatives). Pooled across 5 seeds (paired bootstrap, 5000 metric values), the cascade achieved relaxed *F*_1_-score of 0.338 (95% CI 0.237-0.460) and strict *F*_1_-score of 0.247 (95% CI 0.156-0.351), compared with the oracle 2-stage pipelines 0.455 and 0.338, respectively. The 95% CIs overlap substantially in both modes, but the point estimates fall by roughly 12 (relaxed) and 9 (strict) percentage points relative to the oracle pipeline. The error-attribution profile shifts accordingly: in relaxed mode, the binary stage accounts for 46% (114/249) of false negatives (summed across seeds) and RE for 53%, while NER contributes essentially zero. In strict mode, the binary stage (36%), NER-symptom (29%), and RE (27%) are all material. Although GritLM-SOAP achieves a strong document-classifier mean *F*_1_-score of 72.53% in isolation (Table 2), at the operating threshold used here it misses 26% of ADE-positive test notes when summed across seeds, and these misses are unrecoverable by downstream span extraction. Operating-point selection on the binary stage (eg, a lower decision threshold favoring recall) is therefore a key tuning parameter for any deployed pipeline; full per-seed cascade results are provided in Section S4 in Multimedia Appendix 3.

FollowIR was not designed as a clinical reasoning engine, and its zero-shot performance on this task is competitive with neither the supervised classifiers we trained nor the etiology pipeline. Its inclusion serves to characterize what label-free natural-language baselines can achieve on ADE detection; production-quality ADE detection should not rely on such a model alone.

As an alternative to class weighting, undersampling the majority class was explored and produced comparable but slightly lower performance with similar variance; we selected class weighting to preserve all training data.

### Comparison to Previous Studies

Our model performance compares favorably to other recent attempts in the literature to carry out document-level classification of whether an encounter note contains any instance of ADE. For instance, van de Burgt et al [38] developed an algorithm to detect ADEs in electronic health records and reported a PPV of 13% and sensitivity of 93% for flagging documents containing ADEs. Siegersma et al [18] created a pipeline to flag documents containing ADRs and their best method achieved an *F*_1_-score of 0.67 [19]. In our evaluation, the SOAP Rewrite+ LLM Embeddings Classifier (GritLM-SOAP) achieved a mean *F*_1_-score of 72.53% (95% CI 62.40%‐81.82%) across 5 random seeds, with the BioBERT-SOAP weighted variant reaching the highest mean recall at 76.34% (95% CI 62.69%‐89.66%). These numbers are not directly comparable to the prior studies because of differences in cohort construction, annotation criteria, and evaluation protocol; we report bootstrap CIs to enable the reader to assess the precision of these estimates given our 217-note test set (71 ADE-positive).

### Limitations

The test set contains 217 notes, of which 71 are ADE-positive. At this scale, a difference of 1 percentage point in *F*_1_-score corresponds to approximately one note’s classification differing between models, and reported differences smaller than the bootstrap CIs (Table 2) should not be interpreted as evidence of one model outperforming another. We address this fragility through bootstrap CIs across 5 random seeds in Table 2, with per-seed values and cross-model rankings provided as Multimedia Appendix 3. Future work involving larger annotated cohorts would yield more discriminating comparisons.

We did not perform a systematic hyperparameter search. For the BERT-family classifiers, we set the learning rate and added class weights to address the class imbalance, but we did not tune these or other settings such as batch size, number of epochs, or dropout through a grid or Bayesian search. The baseline models may therefore still have room for improvement with more thorough tuning.

Several interpretative challenges emerged during our qualitative review of the model outputs. First, the LLM tended to prioritize specific diagnoses. Due to privacy and data governance restrictions that prevent disclosure of the actual patient encounter notes, we present an illustrative example: in cases where edema was annotated as an adverse effect of amlodipine, the model consistently ranked “heart failure” and “renal dysfunction” as more probable etiologies than medication side effects. This pattern suggests that LLMs may default to pathophysiological causes over pharmacological ones, particularly when symptoms could have multiple equally plausible explanations. This may limit sensitivity to medication-related adverse events.

The cohort was constructed using a medication-replacement subselection criterion that enriches for clinically actionable ADEs, those significant enough to prompt a prescribing change. ADEs managed via dose adjustment, drug holiday, increased monitoring, or symptomatic treatment without medication change are systematically underrepresented. The trained models should therefore be interpreted as detectors of clinically actionable ADEs that prompt prescribing changes, rather than general-purpose ADE detectors. This framing is appropriate for the screening-pipeline application motivating this work but limits direct generalization to passive ADE surveillance. Studies aiming to detect the broader ADE population would require a subselection criterion not anchored on prescribing changes.

As described in the “Methods” section, the 2 waves differ in their selection with respect to ED contact: Wave 1 was selected for a recent ED visit and is enriched for acute presentations, whereas Wave 2 did not condition on ED contact. Because a primary care population comprises both acute and nonacute presentations, a cohort spanning both waves is in this respect more representative of that setting than either wave alone. We note, however, that ED contact was neither required nor recorded for Wave 2, so its acuity composition is uncharacterized, and the relative proportion of acute to nonacute presentations in the combined cohort reflects how the waves were assembled rather than any specific clinic. Reported performance should therefore be read as reflecting this particular cohort composition and may not transfer cleanly to a setting whose acuity mix differs substantially from ours.

Our evaluation cohort was enriched for ADEs (32.7% prevalence), so the precision reported here should not be expected to hold in an unenriched, real-world deployment setting. If we hold sensitivity and specificity at the values implied by GritLM-SOAP, then at a real-world prevalence below 0.1% the same classifier would produce on the order of 74 true positives and roughly 14,300 false positives per 100,000 encounters, which reduces precision to approximately 0.5%. The precision reported here therefore reflects the enriched evaluation setting and would not transfer to passive, population-wide ADE surveillance. Calculations for the above scenario are detailed in Multimedia Appendix 4.

We acknowledge the limitations of the lack of metrics supporting annotation due to the approach applied. However, the collaborative approach ensured consistent application of annotation criteria and allowed real-time resolution of ambiguous cases, particularly for the clinically nuanced judgments required in ADE identification. Both annotators received training on ADE identification criteria prior to annotation, and a supervising pharmacist was consulted for cases requiring additional clinical expertise.

The training-test split was performed at the note level rather than the patient level. Of 998 unique patients in the cohort, 18 have notes in both splits, corresponding to 18 of 217 (8.3%) test notes and 4 of 71 (5.6%) ADE-positive test notes from “leaked” patients; 2 patients have ADE-positive notes in both splits. We quantified the impact via the sensitivity analysis in the subsection “Sensitivity to Patient-Level Leakage” under “Results” section that reports both pooled and per-seed views. Pooled across 5 seeds, exclusion of leaked notes changed *F*_1_-score by no more than 1.95 percentage points and ROC-AUC by no more than 0.016 across all 9 models, within bootstrap CIs. Per-seed Δ values vary more substantially (up to 22 percentage points spread within a single model), but with mixed direction across seeds, consistent with random test-set sampling rather than systematic patient-level memorization. Nevertheless, a patient-level stratified split would be the methodologically cleanest choice for future work involving larger cohorts where comparable test-set sizes can be retained.

While the SOAP rewriting step was validated through manual audit and prompt revision, larger-model alternatives (eg, higher-parameter generative models) and LLM-as-judge systematic comparison have not been evaluated and are reserved for future work.

The model frequently misclassified notes containing general allergy or medication intolerance history as indicative of an ongoing ADE. It also struggled with cases where ADEs resulted from medications “working too well,” for example, ACE inhibitors causing low blood pressure and dizziness. Although these are technically ADEs, they were excluded from our analysis due to their ambiguous clinical significance and our focus on underreported, clinically meaningful ADEs. These patterns highlight the model’s limitations in distinguishing between historical, incidental, and clinically significant ADEs.

We trained the RE component only on the 284 ADE-positive notes, since ADE-negative notes contain no annotated relations. The model therefore never saw benign drug-symptom co-occurrences during training. In the end-to-end cascade, however, the RE model receives every candidate pair produced by NER, including false-positive pairs from ADE-negative notes. Since it was never exposed to such benign pairs, it may be biased toward predicting an ADE-drug relation where none exists, and this may have contributed to the false-positive rate we observed in the multistage pipeline.

The risk of hallucination can never be completely eliminated when using an LLM to rewrite text. In our qualitative review of 12 note-SOAP pairs, we found no fabricated entities within that sample. This review covered approximately 1.1% of the dataset, and a larger systematic audit would be needed to characterize hallucination across the full corpus. We therefore identify this limited validation sample as a limitation of the present work.

The BERT-family models are also limited by their input length. BERT-base can only take 512 tokens at a time. Only 0.8% (9/1085) of the original notes were longer than this, but 26.4% (286/1085) of the SOAP-rewritten notes were, and these were truncated. The SOAP format puts the assessment and plan at the end of the note, which is where the clinical reasoning and medication changes are usually documented, so truncating these notes may have removed the most informative part for the BERT-SOAP models. GritLM and FollowIR can take 32,000 tokens, so they processed all the notes in full. This means the comparison between the BERT-SOAP models and the long-context models is not entirely fair, as they did not see the same input, and the BERT-SOAP results may understate what these models can actually do on SOAP notes.

Beyond model behavior, several structural challenges also impacted note classification performance. The limited clinical information in brief notes, the extensive use of shorthand, and frequent spelling mistakes complicated both annotation and model interpretation. Furthermore, discrepancies between the training and test sets as well as subtle differences between ADE and non-ADE classes, as illustrated in the ACE inhibitors example, may have posed challenges for model generalizability and consistent labeling.

In this evaluation, the pipeline (mean *F*_1_-score 60.29%) did not outperform the simpler zero-shot FollowIR classifier on the original notes (mean *F*_1_-score 61.71%), so we present it as an exploratory result. The added reasoning steps did not improve on direct prediction here. Even so, its interpretable intermediate outputs (extracted symptoms and ranked etiologies) make it a useful candidate for clinician-facing applications where decision transparency is valued.

### Future Directions

The span-level extraction analysis presented (see the subsection “Span-Level Extraction Performance” under the “Results” section) shows that, at relaxed-span matching, NER on this dataset is essentially solved, while the bottleneck for end-to-end span extraction in the oracle 2-stage setting is RE. When binary classification is included as a gating stage 0 (3-stage cascade), document-level filtering becomes a coequal bottleneck, accounting for 46% of relaxed-mode false negatives. Future work should therefore prioritize both RE modeling, for example, via richer pair representations, larger annotated training sets, or more sophisticated relation-classification objectives, and binary-stage operating-point selection (decision threshold and recall-precision trade-off) appropriate to a pharmacovigilance screening setting.

### Conclusion

This study provides comprehensive benchmarking across multiple NLP paradigms by evaluating both traditional BERT-based models (BioBERT and BlueBERT) and modern LLM approaches. This comparative analysis provides valuable insights into the strengths and limitations of different architectures, and contributes to a more nuanced understanding of ADE detection performance across model types. Our findings suggest that successful ADE detection requires models that approximate the instincts, experience, and clinical reasoning of healthcare providers. While LLMs benefit from vast knowledge bases, their lack of clinical intuition may limit performance. This gap could be narrowed by training on larger, more diverse datasets that better reflect real-world clinical complexity.

## Supplementary material

10.2196/86467 Multimedia Appendix 1 Prompts used in the study.

10.2196/86467 Multimedia Appendix 2 Model hyperparameters.

10.2196/86467 Multimedia Appendix 3 Per-seed model performance, training stability, patient-level leakage sensitivity analyses, and end-to-end cascade pipeline evaluation.

10.2196/86467 Multimedia Appendix 4 Estimated precision at real-world prevalence.

## Abbreviations

ADE: adverse drug event
ADR: adverse drug reaction
ATC: Anatomical Therapeutic Chemical
AUC: area under the curve
BERT: Bidirectional Encoder Representations from Transformers
CPCSSN: Canadian Primary Care Sentinel Surveillance Network
DPIN: Drug Program Information Network
ED: emergency department
EMR: electronic medical record
IR: Information Retrieval
LLM: large language model
MaPCReN: Manitoba Primary Care Research Network
NER: named entity recognition
NLP: natural language processing
PPV: positive predictive value
PR-AUC: precision-recall area under the curve
RE: relation extraction
ROC-AUC: receiver operating characteristic area under the curve
SMM4H: Social Media Mining for Health
SOAP: Subjective-Objective-Assessment-Plan
TREC: Text Retrieval Conference
