# Supplementary material for: Using Natural Language Processing to Identify Adverse Drug Events Characterized by Medication Replacement in Primary Care Electronic Medical Records: Algorithm and Validation Study
Source: J Med Internet Res. 2026 Aug 10;28:e86467. doi: 10.2196/86467 (PMC13456073; doi:10.2196/86467)
Supplement: Supplementary file 1 [file jmir-v28-e86467-s001.docx]

All prompts follow the Mistral-7b-Instruct-v0.1 instruction format: <Encounter Note>'[note text]'</Encounter Note> [INST] [instruction] [/INST]. The model was run with do_sample=True, max_new_tokens=600, and a fixed random seed for reproducibility within each per-seed regeneration of the SOAP rewrites.

## S1. SOAP Rewriting Prompt

Each raw encounter note was rewritten into Subjective-Objective-Assessment-Plan (SOAP) format using the following prompt. The instruction explicitly prohibits inference and constrains rewriting to structural reorganization and unambiguous abbreviation expansion.

**SOAP Rewriting Prompt:** <Encounter Note>'{note}'</Encounter Note> [INST] Rewrite the provided encounter note in Subjective-Objective-Assessment-Plan (SOAP) format. Reorganize the existing content into SOAP structure without adding any information that is not explicitly stated in the note. Do not infer diagnoses, causes, severity, or clinical reasoning that the note does not state. Expand abbreviations and shorthand to standard medical equivalents only when the meaning is unambiguous; if a term is ambiguous, leave it as written. Limit yourself strictly to the information in the note: [/INST]

The SOAP rewrites were regenerated independently for each of the 5 random training seeds used in classifier evaluation, producing five datasets (SOAP_text_seed_<S>.pkl for S in {42, 1337, 2024, 31415, 271828}). All SOAP-variant binary classification results in the revised manuscript use these regenerated rewrites.

## S2. Symptom Extraction Prompt

For the Etiology List Classification pipeline, symptoms were extracted from SOAP-rewritten notes using the following prompt. The instruction targets diseases, disorders, symptoms, and abnormal lab results that the patient actually experienced, excluding speculative or future-risk mentions.

**Symptom Extraction Prompt:** <Encounter Note>'{note}'</Encounter Note> [INST] List only the diseases, disorders (however common or rare), symptoms and abnormal lab results that are ongoing or the patient suffered in the past, mentioned in this encounter note. Exclude any speculation of disorders' or symptoms' or lab results' etiologies. Exclude medication names, exclude medication management information, exclude disorders that may happen in the future, and do not assume diagnoses unless explicitly stated. Provide the medical term for each disorder, symptom or abnormal lab result from the aforementioned list in a separate numbered list, at the end, under the title - 'Extracted disorder, symptom and lab result terms:\n1.': [/INST]

## S3. Etiology Ranking Prompt

For each extracted symptom, Mistral-7b generated a ranked list of etiologies (differential diagnoses). The prompt prioritizes the treating clinician's implicit or explicit assessment and explicitly excludes listing treatments as etiologies.

**Etiology Ranking Prompt:** <Encounter Note>'{note}'</Encounter Note> [INST] Give the numbered list of etiologies for the patient's symptom '{symptom}', in decreasing order of likelihood by giving priority to the doctor's implicit or explicit views. Only answer if etiologies exist provided if the etiology is NOT a treatment of the symptom and the symptom is not an expected side-effect of the treatment, for example, a diuretic should not be listed as an etiology for hypertension or edema because it is a treatment for those symptoms: [/INST]

## S4. FollowIR Binary Classification – Etiology List Pipeline

The top two ranked etiologies for each symptom were passed to FollowIR for binary medication-causation classification. The system prompt establishes the physician role and clarifies that medication ineffectiveness does not constitute an ADE.

**FollowIR Etiology Classification Template:** [INST] You are a physician, whose job is to determine if the following document contains an Adverse Drug Event or not (true/false). Answer using only one word, one of those two choices. Query: Was the symptom {symptom} caused by a medication according to the ranked etiologies in decreasing order of probability? Document: {etiology_text} Relevant (only output one word, either "true" or "false"): [/INST]

Scoring: Log-softmax over the "true" and "false" token logits at the final position. The exponentiated "true" score yields a probability in [0, 1]. Threshold: 0.5.

## S5. FollowIR Binary Classification – Direct Note Classification

For the direct FollowIR classification approach (without the etiology pipeline), the entire encounter note was passed directly as the document with the following template:

**FollowIR Direct Classification Template:** [INST] You are a physician, whose job is to determine if the following document contains an Adverse Drug Event or not (true/false). Answer using only one word, one of those two choices. Query: Does the encounter note contain any instance of Adverse Drug Event? Document: {note} Contains adverse drug event (only output one word, either "true" or "false"): [/INST]

Scoring method identical to 1.4 (log-softmax, threshold 0.5).

## S6. LLM Embeddings Classifier (GritLM)

The embedding-based classifier used GritLM, a generative representational instruction-tuned language model, to extract contextual embeddings (4096-dimensional) of each encounter note. GritLM's parameters were frozen during training to avoid overfitting. The embeddings were passed through a custom 2-layer fully connected neural network (classification head) to produce binary ADE predictions. No task instruction was provided to GritLM; notes were encoded using the default embedding template.

**GritLM Embeddings Classifier — Architecture:** Embedding extraction: GritLM encodes each encounter note with no task instruction (empty instruction string). The embedding template uses the format: <|user|>\n<|embed|>\n followed by the note text. Classification head: Linear(4096, 128) -> ReLU -> BatchNorm -> Dropout(0.6) -> Linear(128, 1) Loss: BCEWithLogitsLoss with pos_weight=1.6 (minority class upweighting) Optimizer: AdamW (lr=0.0005) Training epochs: 5 Batch size: 8 Decision threshold: 0.5
