# Supplementary material for: Using Natural Language Processing to Identify Adverse Drug Events Characterized by Medication Replacement in Primary Care Electronic Medical Records: Algorithm and Validation Study
Source: J Med Internet Res. 2026 Aug 10;28:e86467. doi: 10.2196/86467 (PMC13456073; doi:10.2196/86467)
Supplement: Supplementary file 2 [file jmir-v28-e86467-s002.docx]

All hyperparameters are reported as used in the final experiments. All BERT-family classifiers (BlueBERT and BioBERT, both Original and SOAP variants) were trained with class-weighted CrossEntropy loss and a learning rate reduced from the script default of 5e-5 to 2e-5 for stability. Each configuration was trained across 5 random seeds (42, 1337, 2024, 31415, 271828) to enable bootstrap confidence intervals on test-set performance.

## S1. Train-Test Split

| **Parameter** | **Value** |
| --- | --- |
| Split ratio | 80% train / 20% test |
| Split method | sklearn.model_selection.train_test_split |
| Stratification | By class label (ADE-positive vs. ADE-negative) |
| Random seed (split) | 24121314 (fixed across all experiments) |
| Training seeds (model fitting) | 42, 1337, 2024, 31415, 271828 (5 independent runs) |
| Shuffle | True |
| Split level | Note-level (not patient-level) |
| Total notes | 1,085 (355 ADE-positive, 730 ADE-negative) |
| Training set size | 868 notes (284 ADE-positive) |
| Test set size | 217 notes (71 ADE-positive) |
| Patient-level overlap | 18 patients in both training and test (8.3% of test notes leaked); see Section *Sensitivity to patient-level leakage* |

## S2. BlueBERT (Both Original and SOAP variants, Fine-Tuned)

| **Parameter** | **Value** |
| --- | --- |
| Pre-trained model | bionlp/bluebert_pubmed_mimic_uncased_L-24_H-1024_A-16 (BlueBERT Large) |
| Framework | HuggingFace Transformers (Trainer API) |
| Task | Sequence classification (num_labels=2) |
| Max sequence length | 512 tokens |
| Training epochs | 5 |
| Evaluation strategy | Per epoch |
| Learning rate | 2e-5 (reduced from script default 5e-5 for stability) |
| Batch size | 8 |
| Class weighting | Class-weighted CrossEntropy (weight_neg = 1.0, weight_pos = n_neg / n_pos ≈ 2.06) |
| Optimizer | AdamW (default) |
| Training seeds | 42, 1337, 2024, 31415, 271828 (5 independent runs per variant) |
| Input text | SOAP-rewritten encounter notes (SOAP variant) / Original notes (Original variant) |

## S3. BioBERT (Both Original and SOAP variants, Fine-Tuned)

| **Parameter** | **Value** |
| --- | --- |
| Pre-trained model | dmis-lab/biobert-base-cased-v1.2 |
| Framework | HuggingFace Transformers (Trainer API) |
| Task | Sequence classification (num_labels=2) |
| Max sequence length | 512 tokens |
| Training epochs | 5 |
| Evaluation strategy | Per epoch |
| Learning rate | 2e-5 (reduced from script default 5e-5 for stability) |
| Batch size | 8 |
| Class weighting | Class-weighted CrossEntropy (weight_neg = 1.0, weight_pos = n_neg / n_pos ≈ 2.06) |
| Optimizer | AdamW (default) |
| Training seeds | 42, 1337, 2024, 31415, 271828 (5 independent runs per variant) |
| Input text | SOAP-rewritten encounter notes (SOAP variant) / Original notes (Original variant) |
| Note | With class weighting, BioBERT-Original-weighted recovers from previously reported F1=0 to mean F1 = 59.78% across 5 seeds (per-seed F1 std = 7.06; range 48.92–66.67) |

## S4. FollowIR (Prompt-Based Classification)

| **Parameter** | **Value** |
| --- | --- |
| Model | FollowIR-7B (jinaai/follow-ir-7b) |
| Backbone | Mistral-7B base (broad pretraining including biomedical text) |
| Fine-tuning data | FollowIR training set: TREC annotator narratives from Robust04, Common Core 2017, News21 — general (non-biomedical) TREC collections |
| Capability exploited | Zero-shot binary relevance judgment in response to a natural-language instruction |
| Classification method | Log-softmax over "true"/"false" token logits at final position |
| Decision threshold | 0.5 |
| Batch size | 1 (etiology pipeline) / 16 (direct classification) |
| Tokenizer padding | Left-padded |
| Truncation | True |
| Random seed | Deterministic (model is zero-shot; same outputs across all 5 evaluation seeds) |
| Etiology pipeline: top-k etiologies | 2 (truncated at "3." in ranked list) |
| Note-level aggregation | OR (any symptom > 0.5 -> ADE-positive) |

## S5. Mistral-7b-Instruct-v0.1 (Text Generation)

| **Parameter** | **Value** |
| --- | --- |
| Model | mistralai/Mistral-7B-Instruct-v0.1 |
| Quantization | None (full precision) |
| Sampling | do_sample=True |
| Max new tokens | 600 |
| Random seed | Fixed within each per-seed regeneration of the SOAP rewrites |
| Tasks | SOAP rewriting (revised prompt, see 1.1), symptom extraction, etiology ranking |
| Per-seed regeneration | SOAP rewrites regenerated independently for each of 5 training seeds, producing SOAP_text_seed_<S>.pkl for S in {42, 1337, 2024, 31415, 271828} |

## S6. LLM Embeddings Classifier (GritLM)

| **Parameter** | **Value** |
| --- | --- |
| Model | GritLM-7B (gritlm package, mode=embedding, max_length=2048) |
| Embedding dimension | 4096 |
| Classification head | Linear(4096, 128) -> ReLU -> BatchNorm -> Dropout(0.6) -> Linear(128, 1) |
| Loss function | BCEWithLogitsLoss (pos_weight=1.6 for minority class upweighting) |
| Threshold | 0.5 |
| Optimizer | AdamW |
| Learning rate | 0.0005 |
| Training epochs | 5 |
| Batch size | 8 |
| GritLM parameters | Frozen (only classification head trained) |
| Task instruction | None (empty instruction string) |
| Input text | SOAP-rewritten encounter notes / Original clinical notes |
| Training seeds | 42, 1337, 2024, 31415, 271828 (5 independent runs per variant) |

## S7. Named Entity Recognition (NER)

The NER component identifies Drug and Symptom entity spans in the test notes. We compared BlueBERT and BioBERT backbones; BlueBERT-NER was selected for the end-to-end pipeline based on per-seed performance (Table 8 in the main text).

| **Parameter** | **Value** |
| --- | --- |
| Backbones evaluated | BlueBERT-Large (bionlp/bluebert_pubmed_mimic_uncased_L-24_H-1024_A-16) and BioBERT-Base (dmis-lab/biobert-base-cased-v1.2) |
| Selected backbone for end-to-end pipeline | BlueBERT-Large |
| Architecture | Token classification head over the BERT backbone |
| Tagging scheme | BIO with 2 entity types (Drug, Symptom) |
| Max sequence length | 512 tokens |
| Training epochs | 5 |
| Learning rate | 5e-5 |
| Batch size | 8 |
| Optimizer | AdamW |
| Training data | 868 training notes (both ADE-positive and ADE-negative; ADE-negative notes contribute as sequences containing no entity spans) |
| Evaluation modes | Strict (exact span boundary match) and Relaxed (token-level overlap between predicted and gold spans) |
| Training seeds | 42, 1337, 2024, 31415, 271828 (5 independent runs per backbone) |

## S8. Relation Extraction (RE)

The RE component classifies (Drug, Symptom) entity pairs as ADE-Drug relations. We compared BlueBERT and BioBERT backbones; BioBERT-RE was selected for the end-to-end pipeline based on per-seed AUC performance (Table 11 in the main text).

| **Parameter** | **Value** |
| --- | --- |
| Backbones evaluated | BlueBERT-Large and BioBERT-Base |
| Selected backbone for end-to-end pipeline | BioBERT-Base |
| Architecture | Pair classification head: Drug and Symptom spans within a note marked with special entity-marker tokens; BERT-encoded representation of the marked input passed to a classification head |
| Max sequence length | 512 tokens |
| Training epochs | 5 |
| Learning rate | 1e-5 |
| Batch size | 16 |
| Optimizer | AdamW with linear warmup over the first 10% of training steps |
| Class weighting | Class-weighted CrossEntropy (pos_weight = sqrt(n_neg / n_pos) ≈ 3.74); square-root weighting dampens the strong class imbalance among (Drug, Symptom) pairs while still up-weighting the positive class |
| Training data | 284 ADE-positive training notes (every (Drug, Symptom) pair labelled positive if it appears as an ADE-Drug relation in the BRAT annotation file, negative otherwise; ADE-negative notes excluded as they contribute only trivial empty-relation sets) |
| Training seeds | 42, 1337, 2024, 31415, 271828 (5 independent runs per backbone) |

## S9. End-to-End Pipeline (BlueBERT-NER + BioBERT-RE)

The end-to-end pipeline composes the selected NER and RE components sequentially. For each test note, NER predicts Drug and Symptom spans; we then enumerate all (Drug, Symptom) pairs and pass each to RE for ADE-Drug classification. The output is the set of predicted ADE-Drug pairs per note.

| **Parameter** | **Value** |
| --- | --- |
| NER component | BlueBERT-NER (see Appendix 2.7) |
| RE component | BioBERT-RE (see Appendix 2.8) |
| Composition | NER -> enumerate all (Drug, Symptom) pairs -> RE -> set of predicted ADE-Drug pairs |
| Test set | 71 ADE-positive notes (subset of the 217-note test set; ADE-negative notes excluded as they have no gold relations against which to score) |
| Evaluation modes | Strict and Relaxed (as in Appendix 2.7) |
| Bootstrap CIs | Per-seed test-set bootstrap (model fixed within each row), n_bootstrap = 1000 per seed |
