# Supplementary material for: Using Natural Language Processing to Identify Adverse Drug Events Characterized by Medication Replacement in Primary Care Electronic Medical Records: Algorithm and Validation Study
Source: J Med Internet Res. 2026 Aug 10;28:e86467. doi: 10.2196/86467 (PMC13456073; doi:10.2196/86467)
Supplement: Supplementary file 3 [file jmir-v28-e86467-s003.docx]

This appendix contains supplementary tables referenced in the main manuscript. S1 provides the per-seed cross-model comparison referenced after Table 7 and in the Limitations section. S2 provides the per-model stability tables (per-seed performance for each model with bootstrap CIs and across-seed std). S3 provides the per-seed sensitivity tables (one per model) referenced in Section "Sensitivity to patient-level leakage". S4 provides per-seed and pooled results for the deployment-realistic 3-stage cascade pipeline (GritLM-SOAP + BlueBERT-NER + BioBERT-RE) referenced in the Discussion.

## S1. Per-seed cross-model comparison

For each seed, all nine models evaluated on the same 217-note test set. Per-seed CIs are test-set bootstrap with model fixed (n_bootstrap = 1000). F1, P, R reported as percentages; AUC values in [0,1].

**Table S1.** Per-seed cross-model comparison, seed 42.

| Model | F1 (95% CI) | P (95% CI) | R (95% CI) | ROC-AUC (95% CI) | PR-AUC (95% CI) |
| --- | --- | --- | --- | --- | --- |
| GritLM-SOAP | 77.42 (69.44, 84.52) | 71.43 (61.97, 81.11) | 84.51 (75.30, 92.50) | 0.895 (0.848, 0.937) | 0.817 (0.731, 0.896) |
| GritLM-Original | 70.75 (62.01, 78.53) | 68.42 (57.50, 78.21) | 73.24 (62.32, 82.72) | 0.831 (0.765, 0.887) | 0.765 (0.671, 0.845) |
| BioBERT-SOAP weighted | 78.43 (70.07, 84.85) | 73.17 (63.63, 82.19) | 84.51 (75.44, 92.76) | 0.909 (0.863, 0.949) | 0.856 (0.774, 0.919) |
| BlueBERT-SOAP weighted | 75.52 (67.17, 82.43) | 75.00 (64.79, 84.85) | 76.06 (66.23, 85.00) | 0.895 (0.846, 0.930) | 0.828 (0.749, 0.892) |
| BlueBERT-Original weighted | 68.57 (58.54, 77.22) | 69.57 (58.06, 80.65) | 67.61 (55.88, 78.26) | 0.848 (0.783, 0.903) | 0.775 (0.674, 0.863) |
| BioBERT-Original weighted | 57.72 (47.55, 66.26) | 55.13 (43.83, 65.75) | 60.56 (48.65, 71.67) | 0.757 (0.690, 0.821) | 0.619 (0.507, 0.735) |
| FollowIR-SOAP | 67.90 (59.74, 75.91) | 60.44 (50.55, 70.79) | 77.46 (67.46, 86.08) | 0.812 (0.745, 0.872) | 0.661 (0.550, 0.766) |
| FollowIR-Original | 61.71 (53.25, 69.90) | 51.92 (42.72, 61.12) | 76.06 (65.75, 85.51) | 0.821 (0.760, 0.877) | 0.675 (0.565, 0.781) |

**Table S2.** Per-seed cross-model comparison, seed 1337.

| Model | F1 (95% CI) | P (95% CI) | R (95% CI) | ROC-AUC (95% CI) | PR-AUC (95% CI) |
| --- | --- | --- | --- | --- | --- |
| GritLM-SOAP | 68.79 (60.13, 75.74) | 62.79 (52.47, 72.53) | 76.06 (65.79, 85.25) | 0.826 (0.763, 0.879) | 0.724 (0.622, 0.815) |
| GritLM-Original | 68.70 (58.99, 77.55) | 75.00 (63.63, 86.67) | 63.38 (52.05, 73.97) | 0.809 (0.744, 0.868) | 0.731 (0.628, 0.818) |
| BioBERT-SOAP weighted | 68.71 (59.49, 76.24) | 60.87 (50.00, 70.79) | 78.87 (68.57, 88.24) | 0.823 (0.763, 0.876) | 0.721 (0.617, 0.810) |
| BlueBERT-SOAP weighted | 65.73 (56.69, 74.33) | 65.28 (54.05, 76.39) | 66.20 (55.07, 77.46) | 0.830 (0.772, 0.881) | 0.711 (0.607, 0.810) |
| BlueBERT-Original weighted | 72.34 (63.49, 80.70) | 72.86 (61.54, 82.86) | 71.83 (61.11, 82.35) | 0.868 (0.812, 0.919) | 0.821 (0.738, 0.889) |
| BioBERT-Original weighted | 60.38 (50.34, 68.64) | 54.55 (43.37, 65.00) | 67.61 (56.25, 78.22) | 0.807 (0.744, 0.864) | 0.686 (0.573, 0.783) |
| FollowIR-SOAP | 64.37 (55.21, 72.63) | 54.37 (43.75, 64.55) | 78.87 (68.75, 87.84) | 0.832 (0.774, 0.884) | 0.690 (0.577, 0.788) |
| FollowIR-Original | 61.71 (52.23, 69.57) | 51.92 (41.93, 61.47) | 76.06 (65.71, 85.72) | 0.821 (0.763, 0.871) | 0.675 (0.560, 0.774) |

**Table S3.** Per-seed cross-model comparison, seed 2024.

| Model | F1 (95% CI) | P (95% CI) | R (95% CI) | ROC-AUC (95% CI) | PR-AUC (95% CI) |
| --- | --- | --- | --- | --- | --- |
| GritLM-SOAP | 72.46 (63.25, 80.25) | 74.63 (62.71, 84.93) | 70.42 (58.97, 80.33) | 0.847 (0.785, 0.900) | 0.753 (0.647, 0.859) |
| GritLM-Original | 71.53 (62.77, 79.75) | 74.24 (64.18, 85.97) | 69.01 (58.57, 79.22) | 0.856 (0.803, 0.907) | 0.783 (0.692, 0.863) |
| BioBERT-SOAP weighted | 60.61 (51.95, 69.27) | 53.19 (43.75, 63.20) | 70.42 (59.72, 80.52) | 0.784 (0.724, 0.849) | 0.697 (0.597, 0.795) |
| BlueBERT-SOAP weighted | 70.15 (60.65, 78.75) | 74.60 (63.63, 84.45) | 66.20 (54.79, 76.81) | 0.878 (0.831, 0.921) | 0.790 (0.693, 0.872) |
| BlueBERT-Original weighted | 69.50 (60.42, 77.34) | 70.00 (58.73, 80.49) | 69.01 (58.21, 79.66) | 0.867 (0.813, 0.918) | 0.809 (0.721, 0.880) |
| BioBERT-Original weighted | 48.92 (38.10, 58.82) | 50.00 (38.09, 61.65) | 47.89 (36.23, 59.76) | 0.727 (0.660, 0.797) | 0.602 (0.490, 0.710) |
| FollowIR-SOAP | 58.33 (48.64, 67.03) | 50.52 (40.45, 60.78) | 69.01 (57.74, 79.37) | 0.787 (0.720, 0.851) | 0.667 (0.563, 0.767) |
| FollowIR-Original | 61.71 (52.94, 69.86) | 51.92 (42.45, 61.62) | 76.06 (65.71, 85.34) | 0.821 (0.762, 0.878) | 0.675 (0.560, 0.780) |

**Table S4.** Per-seed cross-model comparison, seed 31415.

| Model | F1 (95% CI) | P (95% CI) | R (95% CI) | ROC-AUC (95% CI) | PR-AUC (95% CI) |
| --- | --- | --- | --- | --- | --- |
| GritLM-SOAP | 72.06 (62.94, 79.48) | 75.38 (64.18, 85.72) | 69.01 (58.33, 79.49) | 0.838 (0.778, 0.892) | 0.773 (0.683, 0.855) |
| GritLM-Original | 70.31 (60.94, 78.74) | 78.95 (68.00, 89.30) | 63.38 (51.61, 75.00) | 0.812 (0.745, 0.876) | 0.745 (0.653, 0.834) |
| BioBERT-SOAP weighted | 68.92 (60.16, 77.13) | 66.23 (56.09, 76.93) | 71.83 (61.64, 81.67) | 0.855 (0.800, 0.905) | 0.735 (0.634, 0.840) |
| BlueBERT-SOAP weighted | 54.55 (42.48, 64.66) | 66.00 (53.06, 79.59) | 46.48 (33.87, 58.73) | 0.793 (0.730, 0.851) | 0.681 (0.565, 0.781) |
| BlueBERT-Original weighted | 53.42 (43.33, 62.50) | 52.00 (41.09, 62.51) | 54.93 (43.48, 65.79) | 0.749 (0.686, 0.809) | 0.600 (0.484, 0.708) |
| BioBERT-Original weighted | 65.22 (55.40, 73.83) | 67.16 (56.06, 77.59) | 63.38 (52.77, 74.25) | 0.840 (0.787, 0.891) | 0.669 (0.560, 0.790) |
| FollowIR-SOAP | 64.60 (55.99, 72.26) | 57.78 (47.83, 67.65) | 73.24 (63.99, 83.55) | 0.821 (0.763, 0.874) | 0.654 (0.540, 0.757) |
| FollowIR-Original | 61.71 (52.56, 70.21) | 51.92 (42.57, 61.17) | 76.06 (65.28, 86.00) | 0.821 (0.758, 0.873) | 0.675 (0.568, 0.781) |

**Table S5.** Per-seed cross-model comparison, seed 271828.

| Model | F1 (95% CI) | P (95% CI) | R (95% CI) | ROC-AUC (95% CI) | PR-AUC (95% CI) |
| --- | --- | --- | --- | --- | --- |
| GritLM-SOAP | 71.94 (62.99, 79.73) | 73.53 (63.08, 83.68) | 70.42 (59.72, 80.60) | 0.839 (0.778, 0.896) | 0.756 (0.668, 0.838) |
| GritLM-Original | 69.93 (61.07, 77.85) | 69.44 (59.21, 80.00) | 70.42 (58.90, 81.08) | 0.829 (0.765, 0.885) | 0.764 (0.674, 0.849) |
| BioBERT-SOAP weighted | 71.05 (61.65, 79.04) | 66.67 (55.70, 76.32) | 76.06 (65.28, 85.34) | 0.859 (0.797, 0.912) | 0.739 (0.616, 0.849) |
| BlueBERT-SOAP weighted | 71.60 (63.16, 78.69) | 63.74 (54.05, 73.42) | 81.69 (72.13, 90.01) | 0.885 (0.839, 0.927) | 0.738 (0.628, 0.856) |
| BlueBERT-Original weighted | 57.93 (46.73, 66.67) | 56.76 (45.45, 67.61) | 59.15 (46.74, 70.32) | 0.764 (0.694, 0.824) | 0.662 (0.555, 0.754) |
| BioBERT-Original weighted | 66.67 (57.97, 74.25) | 61.18 (51.09, 70.73) | 73.24 (62.32, 83.61) | 0.831 (0.774, 0.882) | 0.660 (0.544, 0.776) |
| FollowIR-SOAP | 63.53 (54.90, 71.77) | 54.55 (44.70, 64.15) | 76.06 (65.57, 85.48) | 0.803 (0.737, 0.862) | 0.657 (0.551, 0.766) |
| FollowIR-Original | 61.71 (52.62, 69.61) | 51.92 (41.90, 61.30) | 76.06 (66.66, 86.11) | 0.821 (0.758, 0.876) | 0.675 (0.551, 0.782) |

## S2. Per-model stability across seeds

For each model, performance on each of 5 random seeds with 95% bootstrap CI (test-set resampling, model held fixed; n_bootstrap = 1000). Mean and std rows summarise across seeds. The std answers "how stable is training across seeds"; the per-seed CI answers "how precisely do we know this metric given the test-set size". F1, P, R reported as percentages; AUC values in [0,1].

**Table S1.** Per-seed stability, GritLM-SOAP

| Seed | F1 (95% CI) | P (95% CI) | R (95% CI) | ROC-AUC (95% CI) | PR-AUC (95% CI) |
| --- | --- | --- | --- | --- | --- |
| 42 | 77.42 (69.44, 84.52) | 71.43 (61.97, 81.11) | 84.51 (75.30, 92.50) | 0.895 (0.848, 0.937) | 0.817 (0.731, 0.896) |
| 1337 | 68.79 (60.13, 75.74) | 62.79 (52.47, 72.53) | 76.06 (65.79, 85.25) | 0.826 (0.763, 0.879) | 0.724 (0.622, 0.815) |
| 2024 | 72.46 (63.25, 80.25) | 74.63 (62.71, 84.93) | 70.42 (58.97, 80.33) | 0.847 (0.785, 0.900) | 0.753 (0.647, 0.859) |
| 31415 | 72.06 (62.94, 79.48) | 75.38 (64.18, 85.72) | 69.01 (58.33, 79.49) | 0.838 (0.778, 0.892) | 0.773 (0.683, 0.855) |
| 271828 | 71.94 (62.99, 79.73) | 73.53 (63.08, 83.68) | 70.42 (59.72, 80.60) | 0.839 (0.778, 0.896) | 0.756 (0.668, 0.838) |
| **mean** | **72.53** | **71.55** | **74.08** | **0.849** | **0.764** |
| *std* | *3.10* | *5.12* | *6.42* | *0.027* | *0.034* |

**Table S2.** Per-seed stability, GritLM-Original.

| Seed | F1 (95% CI) | P (95% CI) | R (95% CI) | ROC-AUC (95% CI) | PR-AUC (95% CI) |
| --- | --- | --- | --- | --- | --- |
| 42 | 70.75 (62.01, 78.53) | 68.42 (57.50, 78.21) | 73.24 (62.32, 82.72) | 0.831 (0.765, 0.887) | 0.765 (0.671, 0.845) |
| 1337 | 68.70 (58.99, 77.55) | 75.00 (63.63, 86.67) | 63.38 (52.05, 73.97) | 0.809 (0.744, 0.868) | 0.731 (0.628, 0.818) |
| 2024 | 71.53 (62.77, 79.75) | 74.24 (64.18, 85.97) | 69.01 (58.57, 79.22) | 0.856 (0.803, 0.907) | 0.783 (0.692, 0.863) |
| 31415 | 70.31 (60.94, 78.74) | 78.95 (68.00, 89.30) | 63.38 (51.61, 75.00) | 0.812 (0.745, 0.876) | 0.745 (0.653, 0.834) |
| 271828 | 69.93 (61.07, 77.85) | 69.44 (59.21, 80.00) | 70.42 (58.90, 81.08) | 0.829 (0.765, 0.885) | 0.764 (0.674, 0.849) |
| **mean** | **70.25** | **73.21** | **67.89** | **0.827** | **0.758** |
| *std* | *1.05* | *4.31* | *4.39* | *0.019* | *0.020* |

**Table S3.** Per-seed stability, BioBERT-SOAP weighted.

| Seed | F1 (95% CI) | P (95% CI) | R (95% CI) | ROC-AUC (95% CI) | PR-AUC (95% CI) |
| --- | --- | --- | --- | --- | --- |
| 42 | 78.43 (70.07, 84.85) | 73.17 (63.63, 82.19) | 84.51 (75.44, 92.76) | 0.909 (0.863, 0.949) | 0.856 (0.774, 0.919) |
| 1337 | 68.71 (59.49, 76.24) | 60.87 (50.00, 70.79) | 78.87 (68.57, 88.24) | 0.823 (0.763, 0.876) | 0.721 (0.617, 0.810) |
| 2024 | 60.61 (51.95, 69.27) | 53.19 (43.75, 63.20) | 70.42 (59.72, 80.52) | 0.784 (0.724, 0.849) | 0.697 (0.597, 0.795) |
| 31415 | 68.92 (60.16, 77.13) | 66.23 (56.09, 76.93) | 71.83 (61.64, 81.67) | 0.855 (0.800, 0.905) | 0.735 (0.634, 0.840) |
| 271828 | 71.05 (61.65, 79.04) | 66.67 (55.70, 76.32) | 76.06 (65.28, 85.34) | 0.859 (0.797, 0.912) | 0.739 (0.616, 0.849) |
| **mean** | **69.54** | **64.03** | **76.34** | **0.846** | **0.750** |
| *std* | *6.37* | *7.46* | *5.67* | *0.046* | *0.062* |

**Table S4.** Per-seed stability, BlueBERT-SOAP weighted.

| Seed | F1 (95% CI) | P (95% CI) | R (95% CI) | ROC-AUC (95% CI) | PR-AUC (95% CI) |
| --- | --- | --- | --- | --- | --- |
| 42 | 75.52 (67.17, 82.43) | 75.00 (64.79, 84.85) | 76.06 (66.23, 85.00) | 0.895 (0.846, 0.930) | 0.828 (0.749, 0.892) |
| 1337 | 65.73 (56.69, 74.33) | 65.28 (54.05, 76.39) | 66.20 (55.07, 77.46) | 0.830 (0.772, 0.881) | 0.711 (0.607, 0.810) |
| 2024 | 70.15 (60.65, 78.75) | 74.60 (63.63, 84.45) | 66.20 (54.79, 76.81) | 0.878 (0.831, 0.921) | 0.790 (0.693, 0.872) |
| 31415 | 54.55 (42.48, 64.66) | 66.00 (53.06, 79.59) | 46.48 (33.87, 58.73) | 0.793 (0.730, 0.851) | 0.681 (0.565, 0.781) |
| 271828 | 71.60 (63.16, 78.69) | 63.74 (54.05, 73.42) | 81.69 (72.13, 90.01) | 0.885 (0.839, 0.927) | 0.738 (0.628, 0.856) |
| **mean** | **67.51** | **68.92** | **67.32** | **0.856** | **0.750** |
| *std* | *8.05* | *5.43* | *13.41* | *0.043* | *0.059* |

**Table S5.** Per-seed stability, BlueBERT-Original weighted.

| Seed | F1 (95% CI) | P (95% CI) | R (95% CI) | ROC-AUC (95% CI) | PR-AUC (95% CI) |
| --- | --- | --- | --- | --- | --- |
| 42 | 68.57 (58.54, 77.22) | 69.57 (58.06, 80.65) | 67.61 (55.88, 78.26) | 0.848 (0.783, 0.903) | 0.775 (0.674, 0.863) |
| 1337 | 72.34 (63.49, 80.70) | 72.86 (61.54, 82.86) | 71.83 (61.11, 82.35) | 0.868 (0.812, 0.919) | 0.821 (0.738, 0.889) |
| 2024 | 69.50 (60.42, 77.34) | 70.00 (58.73, 80.49) | 69.01 (58.21, 79.66) | 0.867 (0.813, 0.918) | 0.809 (0.721, 0.880) |
| 31415 | 53.42 (43.33, 62.50) | 52.00 (41.09, 62.51) | 54.93 (43.48, 65.79) | 0.749 (0.686, 0.809) | 0.600 (0.484, 0.708) |
| 271828 | 57.93 (46.73, 66.67) | 56.76 (45.45, 67.61) | 59.15 (46.74, 70.32) | 0.764 (0.694, 0.824) | 0.662 (0.555, 0.754) |
| **mean** | **64.35** | **64.24** | **64.51** | **0.819** | **0.733** |
| *std* | *8.20* | *9.24* | *7.14* | *0.058* | *0.097* |

**Table S6.** Per-seed stability, BioBERT-Original weighted.

| Seed | F1 (95% CI) | P (95% CI) | R (95% CI) | ROC-AUC (95% CI) | PR-AUC (95% CI) |
| --- | --- | --- | --- | --- | --- |
| 42 | 57.72 (47.55, 66.26) | 55.13 (43.83, 65.75) | 60.56 (48.65, 71.67) | 0.757 (0.690, 0.821) | 0.619 (0.507, 0.735) |
| 1337 | 60.38 (50.34, 68.64) | 54.55 (43.37, 65.00) | 67.61 (56.25, 78.22) | 0.807 (0.744, 0.864) | 0.686 (0.573, 0.783) |
| 2024 | 48.92 (38.10, 58.82) | 50.00 (38.09, 61.65) | 47.89 (36.23, 59.76) | 0.727 (0.660, 0.797) | 0.602 (0.490, 0.710) |
| 31415 | 65.22 (55.40, 73.83) | 67.16 (56.06, 77.59) | 63.38 (52.77, 74.25) | 0.840 (0.787, 0.891) | 0.669 (0.560, 0.790) |
| 271828 | 66.67 (57.97, 74.25) | 61.18 (51.09, 70.73) | 73.24 (62.32, 83.61) | 0.831 (0.774, 0.882) | 0.660 (0.544, 0.776) |
| **mean** | **59.78** | **57.60** | **62.54** | **0.792** | **0.647** |
| *std* | *7.06* | *6.66* | *9.48* | *0.049* | *0.036* |

**Table S7.** Per-seed stability, FollowIR-SOAP.

| Seed | F1 (95% CI) | P (95% CI) | R (95% CI) | ROC-AUC (95% CI) | PR-AUC (95% CI) |
| --- | --- | --- | --- | --- | --- |
| 42 | 67.90 (59.74, 75.91) | 60.44 (50.55, 70.79) | 77.46 (67.46, 86.08) | 0.812 (0.745, 0.872) | 0.661 (0.550, 0.766) |
| 1337 | 64.37 (55.21, 72.63) | 54.37 (43.75, 64.55) | 78.87 (68.75, 87.84) | 0.832 (0.774, 0.884) | 0.690 (0.577, 0.788) |
| 2024 | 58.33 (48.64, 67.03) | 50.52 (40.45, 60.78) | 69.01 (57.74, 79.37) | 0.787 (0.720, 0.851) | 0.667 (0.563, 0.767) |
| 31415 | 64.60 (55.99, 72.26) | 57.78 (47.83, 67.65) | 73.24 (63.99, 83.55) | 0.821 (0.763, 0.874) | 0.654 (0.540, 0.757) |
| 271828 | 63.53 (54.90, 71.77) | 54.55 (44.70, 64.15) | 76.06 (65.57, 85.48) | 0.803 (0.737, 0.862) | 0.657 (0.551, 0.766) |
| **mean** | **63.75** | **55.53** | **74.93** | **0.811** | **0.666** |
| *std* | *3.45* | *3.76* | *3.91* | *0.017* | *0.014* |

**Table S8.** Per-seed stability, FollowIR-Original.

| Seed | F1 (95% CI) | P (95% CI) | R (95% CI) | ROC-AUC (95% CI) | PR-AUC (95% CI) |
| --- | --- | --- | --- | --- | --- |
| 42 | 61.71 (53.25, 69.90) | 51.92 (42.72, 61.12) | 76.06 (65.75, 85.51) | 0.821 (0.760, 0.877) | 0.675 (0.565, 0.781) |
| 1337 | 61.71 (52.23, 69.57) | 51.92 (41.93, 61.47) | 76.06 (65.71, 85.72) | 0.821 (0.763, 0.871) | 0.675 (0.560, 0.774) |
| 2024 | 61.71 (52.94, 69.86) | 51.92 (42.45, 61.62) | 76.06 (65.71, 85.34) | 0.821 (0.762, 0.878) | 0.675 (0.560, 0.780) |
| 31415 | 61.71 (52.56, 70.21) | 51.92 (42.57, 61.17) | 76.06 (65.28, 86.00) | 0.821 (0.758, 0.873) | 0.675 (0.568, 0.781) |
| 271828 | 61.71 (52.62, 69.61) | 51.92 (41.90, 61.30) | 76.06 (66.66, 86.11) | 0.821 (0.758, 0.876) | 0.675 (0.551, 0.782) |
| **mean** | **61.71** | **51.92** | **76.06** | **0.821** | **0.675** |
| *std* | *0.00* | *0.00* | *0.00* | *0.000* | *0.000* |

## S3. Per-seed sensitivity to patient-level leakage

For each model, per-seed Full F1/AUC (test-set bootstrap on the 217-note test set, with model fixed) compared to per-seed Clean F1/AUC (test-set bootstrap on the 199-note clean subset, with model fixed); n_bootstrap = 1000 per seed for both. Δ values are per-seed differences (Clean − Full). F1 as percentages; AUC values in [0,1]. The Etiology pipeline is excluded from this section because its FollowIR-driven scoring is largely deterministic and per-seed variance is driven by Mistral generation stochasticity rather than model training; pooled-only sensitivity for the Etiology pipeline is reported in Table 14.

**Table S1.** Per-seed sensitivity, GritLM-SOAP.

| Seed | Full F1 (95% CI) | Clean F1 (95% CI) | Δ F1 | Full AUC (95% CI) | Clean AUC (95% CI) | Δ AUC |
| --- | --- | --- | --- | --- | --- | --- |
| 42 | 77.42 (69.44, 84.52) | 80.30 (72.59, 87.27) | +2.88 | 0.895 (0.848, 0.937) | 0.908 (0.860, 0.952) | +0.013 |
| 1337 | 68.79 (60.13, 75.74) | 69.59 (60.18, 77.50) | +0.80 | 0.826 (0.763, 0.879) | 0.833 (0.772, 0.890) | +0.007 |
| 2024 | 72.46 (63.25, 80.25) | 74.29 (64.15, 82.36) | +1.83 | 0.847 (0.785, 0.900) | 0.857 (0.794, 0.913) | +0.010 |
| 31415 | 72.06 (62.94, 79.48) | 73.30 (65.08, 80.92) | +1.24 | 0.838 (0.778, 0.892) | 0.844 (0.789, 0.899) | +0.006 |
| 271828 | 71.94 (62.99, 79.73) | 74.14 (65.49, 81.61) | +2.20 | 0.839 (0.778, 0.896) | 0.847 (0.789, 0.899) | +0.008 |

**Table S2.** Per-seed sensitivity, GritLM-Original.

| Seed | Full F1 (95% CI) | Clean F1 (95% CI) | Δ F1 | Full AUC (95% CI) | Clean AUC (95% CI) | Δ AUC |
| --- | --- | --- | --- | --- | --- | --- |
| 42 | 70.75 (62.01, 78.53) | 71.12 (62.02, 78.99) | +0.37 | 0.831 (0.765, 0.887) | 0.835 (0.773, 0.895) | +0.004 |
| 1337 | 68.70 (58.99, 77.55) | 69.62 (59.61, 78.46) | +0.92 | 0.809 (0.744, 0.868) | 0.815 (0.751, 0.878) | +0.006 |
| 2024 | 71.53 (62.77, 79.75) | 72.27 (63.15, 80.95) | +0.74 | 0.856 (0.803, 0.907) | 0.867 (0.809, 0.921) | +0.011 |
| 31415 | 70.31 (60.94, 78.74) | 71.18 (61.81, 79.68) | +0.87 | 0.812 (0.745, 0.876) | 0.813 (0.749, 0.873) | +0.001 |
| 271828 | 69.93 (61.07, 77.85) | 70.30 (61.54, 79.10) | +0.37 | 0.829 (0.765, 0.885) | 0.828 (0.765, 0.887) | -0.001 |

**Table S3.** Per-seed sensitivity, BioBERT-SOAP weighted.

| Seed | Full F1 (95% CI) | Clean F1 (95% CI) | Δ F1 | Full AUC (95% CI) | Clean AUC (95% CI) | Δ AUC |
| --- | --- | --- | --- | --- | --- | --- |
| 42 | 78.43 (70.07, 84.85) | 78.95 (71.32, 86.06) | +0.52 | 0.909 (0.863, 0.949) | 0.913 (0.865, 0.955) | +0.004 |
| 1337 | 68.71 (59.49, 76.24) | 68.78 (60.14, 77.13) | +0.0.7 | 0.823 (0.763, 0.876) | 0.822 (0.760, 0.885) | -0.001 |
| 2024 | 60.61 (51.95, 69.27) | 61.62 (53.06, 70.73) | +1.01 | 0.784 (0.724, 0.849) | 0.794 (0.720, 0.864) | +0.010 |
| 31415 | 68.92 (60.16, 77.13) | 70.29 (61.22, 78.52) | +1.37 | 0.855 (0.800, 0.905) | 0.867 (0.810, 0.916) | +0.012 |
| 271828 | 71.05 (61.65, 79.04) | 70.69 (61.54, 78.91) | -0.36 | 0.859 (0.797, 0.912) | 0.861 (0.802, 0.913) | +0.002 |

**Table S4.** Per-seed sensitivity, BlueBERT-SOAP weighted.

| Seed | Full F1 (95% CI) | Clean F1 (95% CI) | Δ F1 | Full AUC (95% CI) | Clean AUC (95% CI) | Δ AUC |
| --- | --- | --- | --- | --- | --- | --- |
| 42 | 75.52 (67.17, 82.43) | 76.84 (68.52, 84.81) | +1.32 | 0.895 (0.846, 0.930) | 0.903 (0.857, 0.943) | +0.008 |
| 1337 | 65.73 (56.69, 74.33) | 66.33 (56.63, 75.00) | +0.60 | 0.830 (0.772, 0.881) | 0.832 (0.769, 0.887) | +0.002 |
| 2024 | 70.15 (60.65, 78.75) | 72.68 (62.90, 81.25) | +2.53 | 0.878 (0.831, 0.921) | 0.886 (0.838, 0.928) | +0.008 |
| 31415 | 54.55 (42.48, 64.66) | 54.99 (42.72, 65.00) | +0.44 | 0.793 (0.730, 0.851) | 0.798 (0.731, 0.856) | +0.005 |
| 271828 | 71.60 (63.16, 78.69) | 71.76 (62.99, 79.55) | +0.16 | 0.885 (0.839, 0.927) | 0.887 (0.839, 0.931) | +0.002 |

**Table S5.** Per-seed sensitivity, BlueBERT-Original weighted.

| Seed | Full F1 (95% CI) | Clean F1 (95% CI) | Δ F1 | Full AUC (95% CI) | Clean AUC (95% CI) | Δ AUC |
| --- | --- | --- | --- | --- | --- | --- |
| 42 | 68.57 (58.54, 77.22) | 69.66 (59.84, 78.15) | +1.09 | 0.848 (0.783, 0.903) | 0.854 (0.792, 0.907) | +0.006 |
| 1337 | 72.34 (63.49, 80.70) | 72.84 (64.28, 81.30) | +0.50 | 0.868 (0.812, 0.919) | 0.867 (0.809, 0.921) | -0.001 |
| 2024 | 69.50 (60.42, 77.34) | 70.06 (60.80, 78.52) | +0.56 | 0.867 (0.813, 0.918) | 0.871 (0.808, 0.921) | +0.004 |
| 31415 | 53.42 (43.33, 62.50) | 53.06 (42.42, 62.86) | -0.36 | 0.749 (0.686, 0.809) | 0.743 (0.669, 0.806) | -0.006 |
| 271828 | 57.93 (46.73, 66.67) | 57.99 (48.33, 67.18) | +0.06 | 0.764 (0.694, 0.824) | 0.760 (0.691, 0.827) | -0.004 |

**Table S6.** Per-seed sensitivity, BioBERT-Original weighted.

| Seed | Full F1 (95% CI) | Clean F1 (95% CI) | Δ F1 | Full AUC (95% CI) | Clean AUC (95% CI) | Δ AUC |
| --- | --- | --- | --- | --- | --- | --- |
| 42 | 57.72 (47.55, 66.26) | 59.80 (49.15, 69.34) | +2.08 | 0.757 (0.690, 0.821) | 0.767 (0.690, 0.834) | +0.010 |
| 1337 | 60.38 (50.34, 68.64) | 61.49 (51.56, 70.35) | +1.11 | 0.807 (0.744, 0.864) | 0.822 (0.761, 0.880) | +0.015 |
| 2024 | 48.92 (38.10, 58.82) | 50.34 (39.28, 60.61) | +1.42 | 0.727 (0.660, 0.797) | 0.734 (0.658, 0.804) | +0.007 |
| 31415 | 65.22 (55.40, 73.83) | 66.51 (56.55, 75.59) | +1.29 | 0.840 (0.787, 0.891) | 0.854 (0.798, 0.903) | +0.014 |
| 271828 | 66.67 (57.97, 74.25) | 68.38 (59.20, 76.60) | +1.71 | 0.831 (0.774, 0.882) | 0.844 (0.791, 0.898) | +0.013 |

**Table S7.** Per-seed sensitivity, FollowIR-SOAP.

| Seed | Full F1 (95% CI) | Clean F1 (95% CI) | Δ F1 | Full AUC (95% CI) | Clean AUC (95% CI) | Δ AUC |
| --- | --- | --- | --- | --- | --- | --- |
| 42 | 67.90 (59.74, 75.91) | 69.80 (61.64, 77.71) | +1.90 | 0.812 (0.745, 0.872) | 0.827 (0.763, 0.891) | +0.015 |
| 1337 | 64.37 (55.21, 72.63) | 66.16 (56.94, 74.56) | +1.79 | 0.832 (0.774, 0.884) | 0.839 (0.778, 0.892) | +0.007 |
| 2024 | 58.33 (48.64, 67.03) | 59.31 (49.63, 67.80) | +0.98 | 0.787 (0.720, 0.851) | 0.794 (0.724, 0.859) | +0.007 |
| 31415 | 64.60 (55.99, 72.26) | 66.06 (57.53, 74.32) | +1.46 | 0.821 (0.763, 0.874) | 0.828 (0.770, 0.882) | +0.007 |
| 271828 | 63.53 (54.90, 71.77) | 64.02 (54.93, 72.53) | +0.49 | 0.803 (0.737, 0.862) | 0.805 (0.733, 0.870) | +0.002 |

**Table S8.** Per-seed sensitivity, FollowIR-Original.

| Seed | Full F1 (95% CI) | Clean F1 (95% CI) | Δ F1 | Full AUC (95% CI) | Clean AUC (95% CI) | Δ AUC |
| --- | --- | --- | --- | --- | --- | --- |
| 42 | 61.71 (53.25, 69.90) | 63.13 (53.95, 71.43) | +1.42 | 0.821 (0.760, 0.877) | 0.837 (0.774, 0.894) | +0.016 |
| 1337 | 61.71 (52.23, 69.57) | 63.00 (53.90, 71.27) | +1.29 | 0.821 (0.763, 0.871) | 0.835 (0.770, 0.890) | +0.014 |
| 2024 | 61.71 (52.94, 69.86) | 63.25 (54.29, 72.29) | +1.54 | 0.821 (0.762, 0.878) | 0.838 (0.779, 0.892) | +0.017 |
| 31415 | 61.71 (52.56, 70.21) | 63.36 (54.77, 71.43) | +1.65 | 0.821 (0.758, 0.873) | 0.838 (0.783, 0.890) | +0.017 |
| 271828 | 61.71 (52.62, 69.61) | 63.10 (53.62, 70.97) | +1.39 | 0.821 (0.758, 0.876) | 0.837 (0.774, 0.891) | +0.016 |

## S4. Three-stage cascade pipeline (GritLM-SOAP + BlueBERT-NER + BioBERT-RE)

Per-seed and pooled results for the deployment-realistic 3-stage cascade discussed in the main text. All 217 test notes are scored against gold; notes classified as ADE-negative by Stage 0 contribute zero predicted relations and their gold pairs become false negatives in the new "binary-filtered" attribution category. P, R, F1 reported as decimal fractions in [0,1]; n_bootstrap = 1000 per seed; pooled CIs from paired bootstrap (5 seeds × 1000 = 5000 metric values).

**Table S1.** Stage 0 (GritLM-SOAP) confusion per seed on the 217-note test set.

| Seed | TP | FP | TN | FN | Precision | Recall | F1 |
| --- | --- | --- | --- | --- | --- | --- | --- |
| 42 | 60 | 24 | 122 | 11 | 0.714 | 0.845 | 0.774 |
| 1337 | 54 | 32 | 114 | 17 | 0.628 | 0.761 | 0.688 |
| 2024 | 50 | 17 | 129 | 21 | 0.746 | 0.704 | 0.725 |
| 31415 | 49 | 16 | 130 | 22 | 0.754 | 0.690 | 0.721 |
| 271828 | 50 | 18 | 128 | 21 | 0.735 | 0.704 | 0.719 |

**Table S2.** Cascade end-to-end strict span-match performance per seed (test-set bootstrap, model fixed).

| Seed | F1 (95% CI) | Precision (95% CI) | Recall (95% CI) |
| --- | --- | --- | --- |
| 42 | 0.301 (0.220, 0.383) | 0.238 (0.170, 0.317) | 0.408 (0.303, 0.523) |
| 1337 | 0.237 (0.169, 0.318) | 0.171 (0.116, 0.237) | 0.388 (0.278, 0.515) |
| 2024 | 0.272 (0.186, 0.357) | 0.224 (0.146, 0.315) | 0.347 (0.242, 0.451) |
| 31415 | 0.221 (0.152, 0.286) | 0.168 (0.114, 0.225) | 0.327 (0.219, 0.442) |
| 271828 | 0.204 (0.137, 0.277) | 0.149 (0.098, 0.211) | 0.327 (0.218, 0.444) |

**Table S3.** Cascade end-to-end relaxed span-match performance per seed (test-set bootstrap, model fixed).

| Seed | F1 (95% CI) | Precision (95% CI) | Recall (95% CI) |
| --- | --- | --- | --- |
| 42 | 0.406 (0.322, 0.491) | 0.321 (0.247, 0.410) | 0.551 (0.435, 0.670) |
| 1337 | 0.325 (0.250, 0.396) | 0.234 (0.172, 0.302) | 0.531 (0.413, 0.650) |
| 2024 | 0.368 (0.279, 0.456) | 0.303 (0.217, 0.399) | 0.469 (0.354, 0.581) |
| 31415 | 0.291 (0.220, 0.364) | 0.220 (0.164, 0.290) | 0.429 (0.312, 0.561) |
| 271828 | 0.300 (0.230, 0.373) | 0.219 (0.162, 0.285) | 0.480 (0.360, 0.600) |

**Table S4.** Pooled cascade vs oracle 2-stage pipeline (paired bootstrap, 5 seeds × 1000 = 5000 metric values).

| Pipeline | Mode | F1 (95% CI) | Precision (95% CI) | Recall (95% CI) |
| --- | --- | --- | --- | --- |
| 2-stage (oracle, n=71 ADE+ notes) | strict | 0.338 (0.236, 0.465) | 0.272 (0.174, 0.419) | 0.457 (0.344, 0.573) |
| 2-stage (oracle, n=71 ADE+ notes) | relaxed | 0.455 (0.358, 0.583) | 0.365 (0.264, 0.522) | 0.618 (0.490, 0.750) |
| 3-stage cascade (n=217 test notes) | strict | 0.247 (0.156, 0.351) | 0.190 (0.112, 0.294) | 0.359 (0.237, 0.490) |
| 3-stage cascade (n=217 test notes) | relaxed | 0.338 (0.237, 0.460) | 0.259 (0.171, 0.387) | 0.492 (0.343, 0.642) |

**Table S5.** Cascade error attribution by mode (false negatives summed across seeds).

| Mode | Total FN | Binary-filtered | NER-drug | NER-symptom | NER-both | RE-pair |
| --- | --- | --- | --- | --- | --- | --- |
| strict | 314 | 114 (36%) | 15 (5%) | 92 (29%) | 9 (3%) | 84 (27%) |
| relaxed | 249 | 114 (46%) | 0 (0%) | 4 (2%) | 0 (0%) | 131 (53%) |
