# Supplementary material for: Using Natural Language Processing to Identify Adverse Drug Events Characterized by Medication Replacement in Primary Care Electronic Medical Records: Algorithm and Validation Study
Source: J Med Internet Res. 2026 Aug 10;28:e86467. doi: 10.2196/86467 (PMC13456073; doi:10.2196/86467)
Supplement: Supplementary file 4 [file jmir-v28-e86467-s004.docx]

This appendix contains calculations used to determine real-world precision.

**Step 1 -** Calculate specificity (Sp) from the reported metrics.

Definition of positive predictive value:

PPV = (Sn * P) / (Sn * P + (1 - Sp) * (1 - P))

Rearranged to isolate Sp:

Sp = 1 - (Sn * P * (1 - PPV)) / (PPV * (1 - P))

Substituting Sn = 0.7408, P = 0.327, PPV = 0.7155:

Sp = 1 - (0.7408 * 0.327 * (1 - 0.7155)) / (0.7155 * (1 - 0.327))

Sp = 1 - (0.06892 / 0.48153)

Sp = 1 - 0.1431 = 0.8569 (approx. 85.7%)

**Step 2 -** Apply the fixed Sn and Sp at deployment prevalence.

TP = N * P' * Sn

FP = N * (1 - P') * (1 - Sp)

PPV' = TP / (TP + FP)

Substituting N = 100000, P' = 0.001, Sn = 0.7408, Sp = 0.8569:

TP = 100000 * 0.001 * 0.7408 = 74.1

FP = 100000 * 0.999 * 0.1431 = 14296

PPV' = 74.1 / (74.1 + 14296) = 0.0052 (approx. 0.5%)

Where: Sn = sensitivity (recall), Sp = specificity, P = cohort prevalence,

P' = deployment prevalence, N = number of encounters, PPV = precision.
